# Biocatalyzed Redox Processes Employing Green Reaction Media

**DOI:** 10.3390/molecules25133016

**Published:** 2020-07-01

**Authors:** Carmen Aranda, Gonzalo de Gonzalo

**Affiliations:** 1 Institute of Natural Resources and Agrobiology of Seville (IRNAS), CSIC, 41012 Seville, Spain; mcaranda@irnas.csic.es; 2 Organic Chemistry Department, University of Seville, 41012 Seville, Spain

**Keywords:** biocatalysis, deep eutectic solvents, biobased solvents, neat conditions, sustainable processes

## Abstract

The application of biocatalysts to perform reductive/oxidative chemical processes has attracted great interest in recent years, due to their environmentally friendly conditions combined with high selectivities. In some circumstances, the aqueous buffer medium normally employed in biocatalytic procedures is not the best option to develop these processes, due to solubility and/or inhibition issues, requiring biocatalyzed redox procedures to circumvent these drawbacks, by developing novel *green* non-conventional media, including the use of biobased solvents, reactions conducted in neat conditions and the application of neoteric solvents such as deep eutectic solvents.

## 1. Introduction

Biocatalysis can be defined in a broad sense as the mediation of chemical reactions by means of biological systems, including isolated enzymes, whole cells or cell-free extracts [1]. As recently stated by Sheldon and Brady [2], biocatalysis agrees with ten of the twelve principles of green chemistry, thus being the most sustainable and environmentally friendly approach for the preparation of high added value molecules [3,4,5], even at industrial scale [6,7].

Among the six different types of biocatalysts according to the type of reaction they catalyze, oxidoreductases (EC 1) are, together with hydrolases, the most employed family of enzymes with synthetic purposes. This large group of enzymes, with 22 types of oxidoreductase subtypes, are involved in all type of redox reactions, catalyzing oxidation and reductions processes [8,9,10,11]. The application of oxidoreductases to obtain valuable building blocks is a current area of interest in organic synthesis, due to the mild reaction conditions and oxidants/reductants required for their function, combined with the generally exquisite stereoselectivity afforded by this type of catalysts.

Water is the natural medium in which enzymes perform their activity. Due to its unique properties, water can be considered an environmentally friendly solvent, which represents an advantage over other solvents, but sometimes its use in organic syntheses catalyzed by enzymes presents some drawbacks. Firstly, water is a very high polar solvent, so the application of enzymes for synthetic purposes is often hindered by the low solubility of many organic substrates and products in aqueous buffers, leading to substrate concentration restrictions [12]. In addition, water can lead to undesired side reactions (hydrolysis), which also leads to lower yields than in absence of water.

In order to circumvent these problems, different non-conventional media have been applied in biocatalysis, since the discovery in the 1980s [13,14] that biocatalysts can perform their activity in organic solvents [15], with some advantages over the use of aqueous medium. Thus, organic solvents have been widely exploited in reaction catalyzed by enzymes, but it has to be considered that their application presents several environmental disadvantages, due to their inherent toxicity and the problems derived from their flammability or explosion risks. For these reasons, green solvents approaches are required [16] in order to minimize the negative impact of the solvent media in the *E* factor of the biocatalytic process [17]. One way to reduce the toxicity of organic (co)solvents has been developed by using biobased solvents [18,19,20,21], that is, solvents obtained from natural renewable sources which present unique characteristics. Typical examples shown in this review are cyclopentyl methyl ether (CPME), as well as 2-methyltetrahydrofuran (2-MeTHF), which are well established as valuable (co)solvents in several biotransformations, including those catalyzed by redox enzymes. Moreover, Guajardo and Domínguez de María have also demonstrated last year that Cyrene^TM^ is a promising biosolvent in lipase-catalyzed transformations [22], but the development of more environmentally friendly reaction media is not only circumscribed to organic solvents, as other solutions have also been applied. Thus, supercritical solvents (CO_2_) have appeared as a promising alternative to organic solvents [23,24,25], but almost all the examples using liquid CO_2_ in biocatalyzed processes have been performed in reactions using hydrolases. Matsuda et al. have recently started to use CO_2_-expanded biobased liquids as a green alternative for lipase-catalyzed biotransformations [26]. Finally, neoteric solvents have appeared as valuable reaction media. Thus, several reviews cover the application of ionic liquids (ILs) as (co)solvents in biocatalysis [27,28,29,30]. These compounds are salts containing poorly coordinated ions that are liquid below 100 °C, normally at room temperature, the so-called room temperature ionic liquids (RTILs). RTILs have been used for biocatalysis purposes since the 2000s. They present low or no vapor pressure, low flammability, thermal robustness and are able to stabilize enzymes and solubilize most of the substrates. But RTILs presents some drawbacks, due to their environmental effect, accumulation in the medium and toxicity. By this reason, a more environmentally approach has been developed in the last few times by using deep eutectic solvents (DESs), compounds formed by the combination of a hydrogen-bond donor with a hydrogen-bond acceptor [31,32,33].

In the present review, all the efforts performed to develop biocatalyzed redox processes using green reaction media will be summarized. Thus, different oxidative and reductive biotransformations carried out in neat conditions, as well of those developed in aqueous systems containing biobased solvents or DESs will be analyzed.

## 2. Biocatalyzed Redox Processes at Neat Conditions

An attractive approach towards sustainable biocatalytic processes is to carry out enzymatic reactions directly in the substrate(s) without the addition of any solvent, since high productivity, competitiveness and reduced waste can be achieved [12]. There are several solvent-free systems including redox enzymes described in literature, using either whole-cells or isolated biocatalysts [34,35,36,37]. In all these examples, both the immobilization of isolated enzymes and/or the addition of a small amount of water (‘structural water’) were required to maintain the activity and flexibility of the biocatalysts. In some cases, the hydrophility of the (co)substrate showed a strong effect on enzyme catalysis, causing the stripping of the essential enzyme-bound water [38].

Alcohol dehydrogenases (ADHs) are oxidoreductases that catalyze the reversible reaction between alcohols and carbonyl compounds (aldehydes and ketones) through a redox process. In order to achieve these reactions, a nicotinamide cofactor, NAD(P)H, is required to perform the electron transfer from/or to the substrate or electron source [39,40,41]. ADHs can be employed as free enzymes or as whole-cells systems, which present some advantages as they are cheaper than isolated enzymes, easier to handle and present an efficient internal cofactor regeneration system. In contrast, lower yields and selectivities can be obtained due to competitive processes in the cell system and solubility and productivity issues can also occur. Whole-cells expressing ADHs have been demonstrated to work on micro-aqueous systems in organic solvents [42] and neat substrates [43] for the reduction of a series of ketones.

**Scheme 1:**
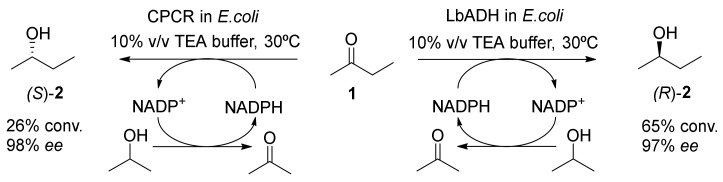
Enantioselective biocatalytic synthesis of (*R*)- and (*S*)-2-butanol with *E. coli* cells expressing ADHs in a continuous micro-aqueous solvent-free reaction system.

The applicability of ADHs in neat substrate systems has also been proved in the enantioselective synthesis of 2-butanol (**2**) in which a continuous micro-aqueous system was applied to minimize the time-dependent racemization of the process (Scheme 1) [44]. In this case, the reaction medium consisted on a mixture of the substrate 2-butanone (**1**) and the 2-propanol used for NADPH cofactor regeneration. The system was tested with two stereocomplementary enzymes expressed in *Escherichia coli*, the ADH from *Lactobacillus brevis* (LbADH) and from *Candida parapsilosis* (CPCR), enabling the production of both enantiomers (*R*)- and (*S*)-**2**, respectively. Optimizing the flux and so the residence time, the enantiomeric excess (*ee*) of the product could be increased while reducing the substrate conversion.

On the one hand, *E. coli* cells expressing LbADH yielded the (*R*)-**2** with a 65% conversion starting with 0.1 M of ketone **1,** attaining a 97% *ee* and high space-time yields of 2278 g/L day. On the other hand, using CPCR a 26% conversion of (*S*)-**2** with 98% *ee* was achieved from 5.0 M of **1**, leading to a space-time yield of 461 g/L day.

The use of isolated biocatalysts was also tried in the reduction of **1** in neat substrate by using the ADH evo-1.1.200 and 1,4-butanediol (1,4-BD) as a sacrificial electron donor [38]. However, although a linear product formation of **2** over 10 days was observed, very low conversion rates (<1%) were achieved. This fact was attributed to the high hydrophility of 1,4-BD making the enzyme to lose flexibility and more apolar solvents, such as methyl *tert*-butyl ether and toluene, were required to achieve higher conversions.

**Scheme 2:**
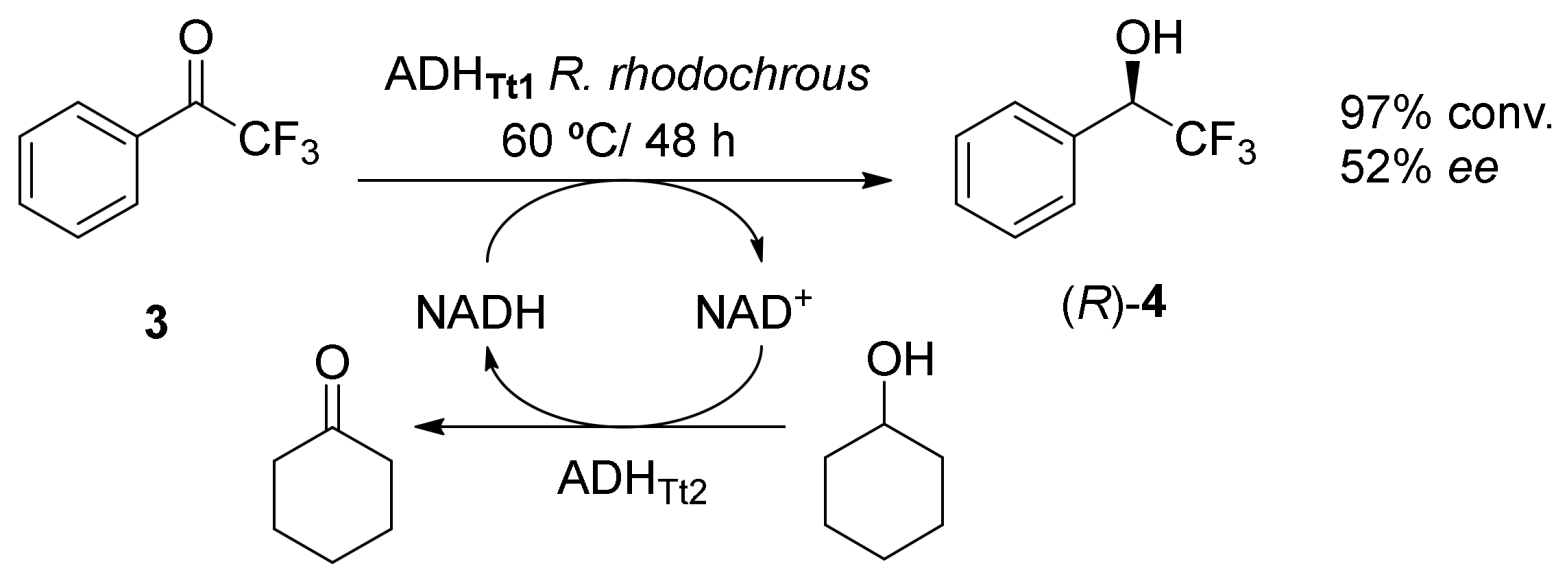
Whole-cell bioconversion of 2,2,2-trifluoroacetophenone (**3**) to α-(trifluoromethyl) benzyl alcohol (*R*)-**4** using ADHs and cyclohexanol oxidation for cofactor recycling.

*Rhodococcus rhodochrous* cells harboring ADHs were also proved to work in neat substrate as reported in the reduction of 2,2,2-trifluoroacetophenone (**3**) to α-(trifluoromethyl)benzyl alcohol (**4**), using cyclohexanol (1:1 *v*/*v*) for cofactor regeneration (Scheme 2) [45]. In this case, the expression of a thermophile enzyme enabled the heat-pretreatment of cells and the performance of reactions at 60 °C, resulting higher conversions due to an increase in the membrane permeability and the inactivation of other indigenous enzymes. Overall, high product concentration (3.6 M) and productivity (190 mol **4**/kg cells h) were achieved in 48 h, although with low stereoselectivity (52%) in the formation of (*R*)-**4**.

Besides bioreduction processes catalyzed by ADHs, other reactions employing neat conditions have been developed employing oxidative enzymes. Thus, recent examples with the newly discovered fungal unspecific peroxygenases (UPOs) have also been reported by Hollmann’s group [46,47]. UPOs are promising enzymes able to perform a wide set of oxyfunctionalization reactions acting as ‘self-sufficient’ monooxygenases only requiring H_2_O_2_ for activation [48,49]. These enzymes are functionally similar to the well-known P450-monooxygenases, but their higher stability due to their extracellular nature and the non-necessity of expensive cofactors and auxiliary enzymes for catalysis, make peroxygenases more attractive for synthetic chemistry. However, the dosage of H_2_O_2_ is one of the limiting factors in the peroxygenase-catalyzed reactions, since a high concentration of this oxidant can inactivate the biocatalysts. For that reason, the peroxide needs to be continuously supplied or *in situ* generated to keep its concentration in limits enough to get a good activity without negative impact in their stability [50,51]. UPOs present also the advantage of being able to work with organic peroxides as co-substrates, thus expanding their catalysis to non-aqueous reaction media. These extracellular enzymes required immobilization to be applied as isolated biocatalysts and the low activity recovery after this procedure is the major challenge on this kind of processes.

**Scheme 3:**
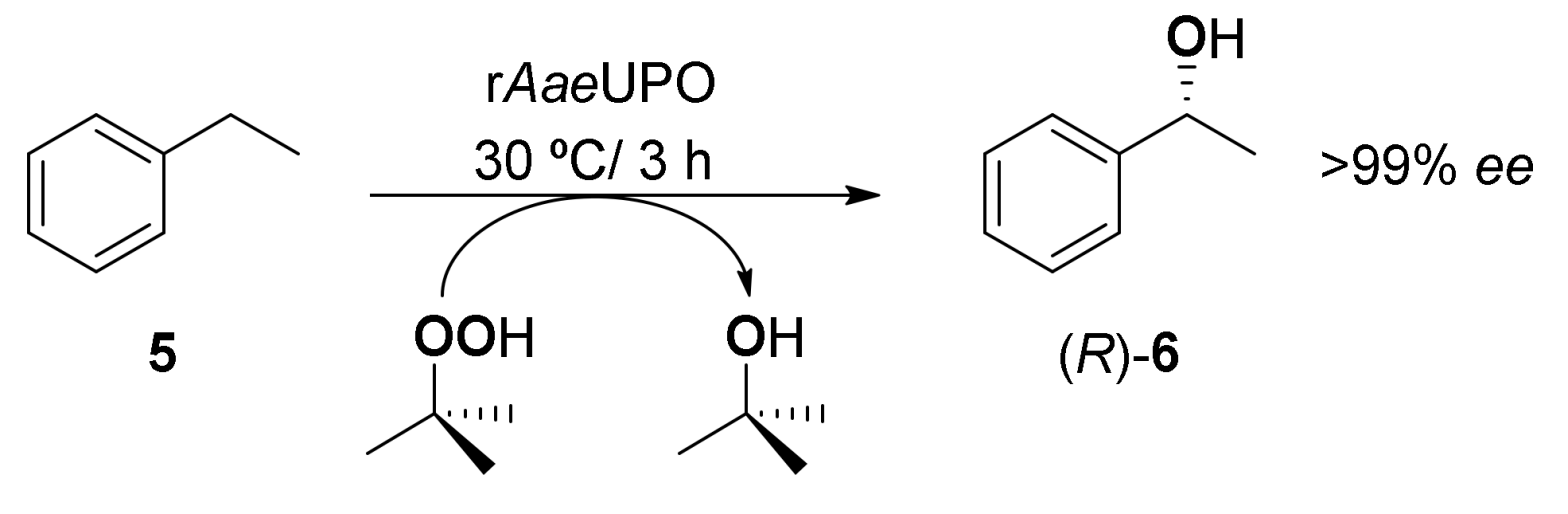
Stereoselective hydroxylation of ethylbenzene to (*R*)-phenylethanol using the recombinant peroxygenase from *Agrocybe aegerita* (r*Aae*UPO) and *tert*-butyl hydroperoxide in neat substrate.

The first evidence of the use of peroxygenases in neat conditions was in the selective hydroxylation of ethylbenzene (**5**) using the recombinant peroxygenase from *Agrocybe aegerita* (r*Aae*UPO) (Scheme 3) [46]. *tert*-Butyl hydroperoxide (*^t^*BuOOH) was used as co-substrate and oxygen donor due to the higher solubility in hydrophobic media, and was found to be a milder oxidant compared to H_2_O_2_. The system was demonstrated on semipreparative scale (250 mL) yielding 1.25 g of the enantioenriched (*R*)-phenylethanol (**6**, 98% *ee*) within 3 h of reaction where the *^t^*BuOOH was continuously supplied to avoid enzyme inactivation. Under these conditions, authors claimed that the biocatalyst was able to perform 90,000 total turnover numbers (TTN).

**Scheme 4:**
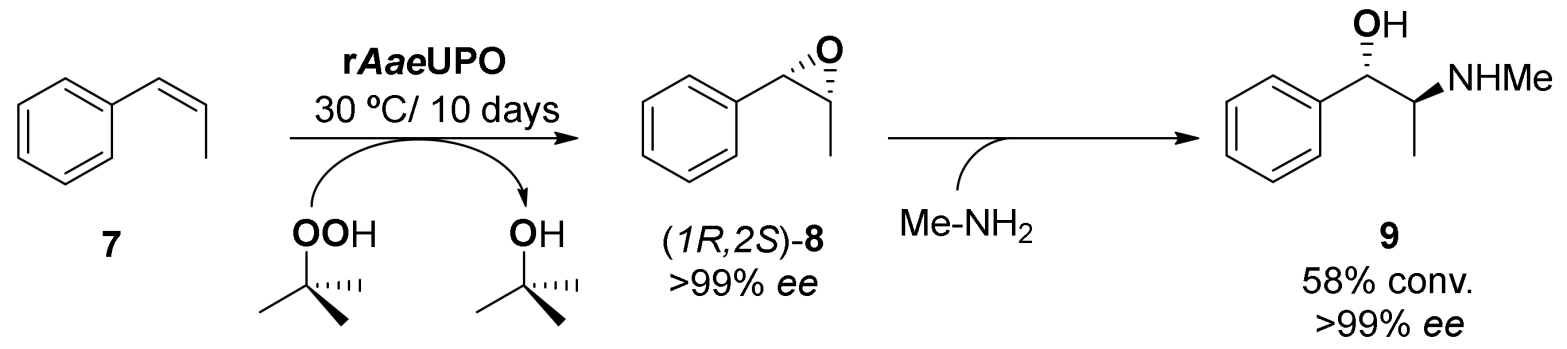
Chemoenzymatic process for the synthesis of (pseudo)ephedrine starting by enzymatic epoxidation of cis-β-methylstyrene in neat substrate and following by the spontaneous oxirane ring-opening employing methylamine.

The same enzyme was later proved to selectively epoxidize a set of styrene derivatives in the neat substrates [47]. Thus, it was applied to build an interesting chemoenzymatic process to afford the valuable drug (pseudo)ephedrine **9** (Scheme 4). A first epoxidation step of *cis*-β-methylstyrene (**7**), with a high stereoselectivity (>99%) and product concentration (360 mM), yielded (*1R,2S*)-**8**, that was chromatographically purified. The oxirane-ring was chemically opened with methyl amine to form the desired compound **9** with a good overall yield (58%).

## 3. Application of Bio-Based Solvents in Redox Reactions Catalyzed by Enzymes

The ideal implementation of solvent-free chemical processes is not always possible and usually (co)solvents are needed to improve mass and heat transfer, reaction rates, selectivities or even the position of the chemical equilibria. However, the concern about their high environmental impact and waste generation is driving the attention to search for greener alternatives, such as solvents derived from biomass. In this context, cyclopentyl methyl ether (CPME), 2-methyltetrahydrofurane (2-MeTHF) and other solvents have emerged as promising ecofriendly solution in redox enzyme catalysis, replacing other traditional solvents in biocatalytic processes [18,19,20,21]. It has to be considered that these solvents present nowadays some drawbacks, as their high price and scarce availability, a not assessed toxicology and that they are still unknown solvents with a lack of data about them. Bio-based solvents have been widely employed with hydrolases, but only examples with CPME and 2-MeTHF have been found in reactions catalyzed by oxidoreductases.

**Figure 1:**
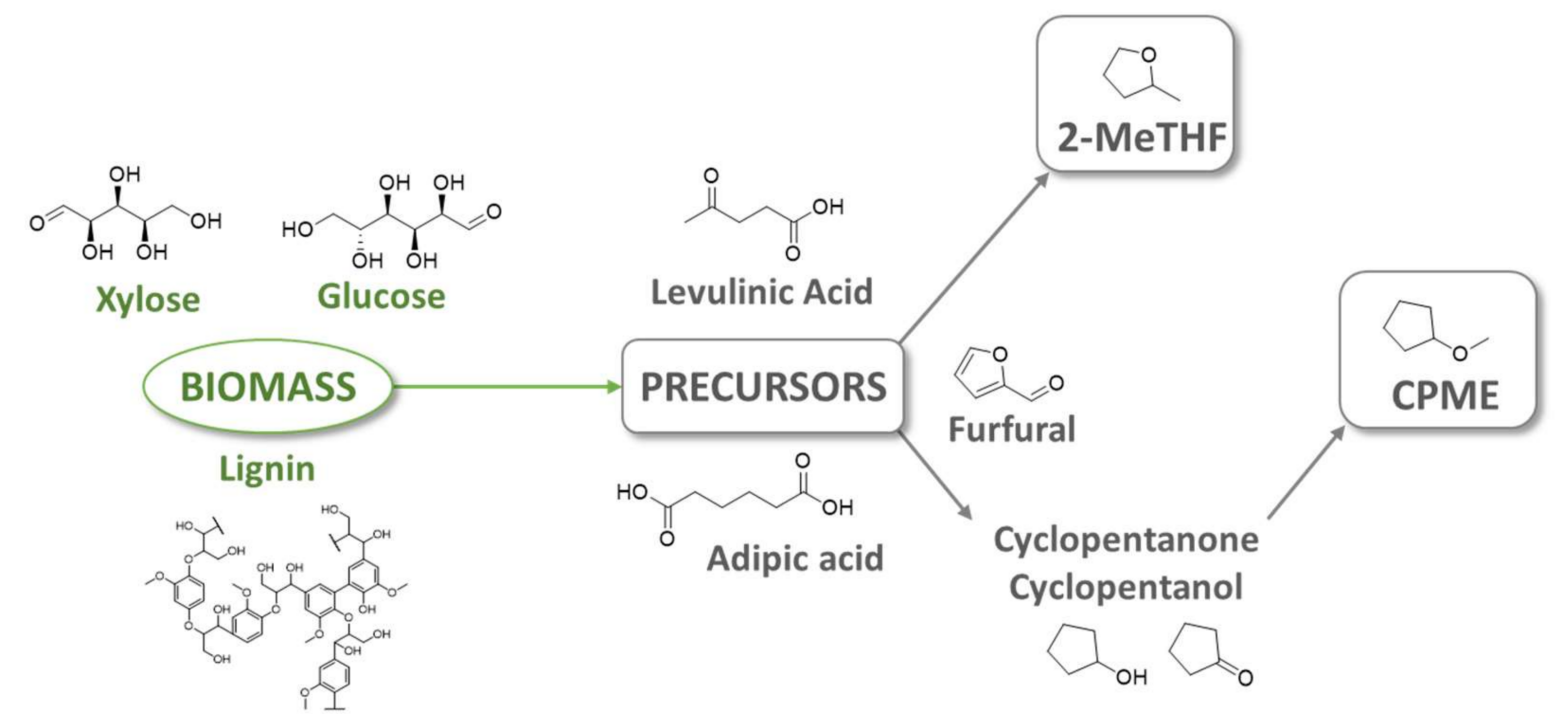
Biogenic pathways options for the synthesis of bio-based CPME and 2-MeTHF.

Very recently, the suitability of CPME as solvent for biotransformations has been pointed out. This solvent presents advantages such as a low solubility in water (1.1 g CPME/100 g) allowing two liquid phase systems, low toxicity and negligible peroxide formation rate. Moreover, it presents manageable boiling point (106 °C), narrow explosion ranges and stability to harsh acid or basic reaction conditions. Although nowadays the synthesis of CPME is based on petrochemicals, a biogenic synthetic pathway could be contemplated through the use of biosynthesized precursors (cyclopentanone or cyclopentanol) obtained from biomass-derived furfural or adipic acid (Figure 1) [19,52].

However, these biogenic routes do not assure a sustainable process in the synthesis of these solvents and life-cycle assessment (LCA) are necessary to study the impact of each particular example. In addition, it has to be taken into account that processes performed in mixtures CPME/aqueous buffer occur in biphasic systems, which can present some inconveniences due to mass transfer issues between the two reaction phases.

**Scheme 5:**
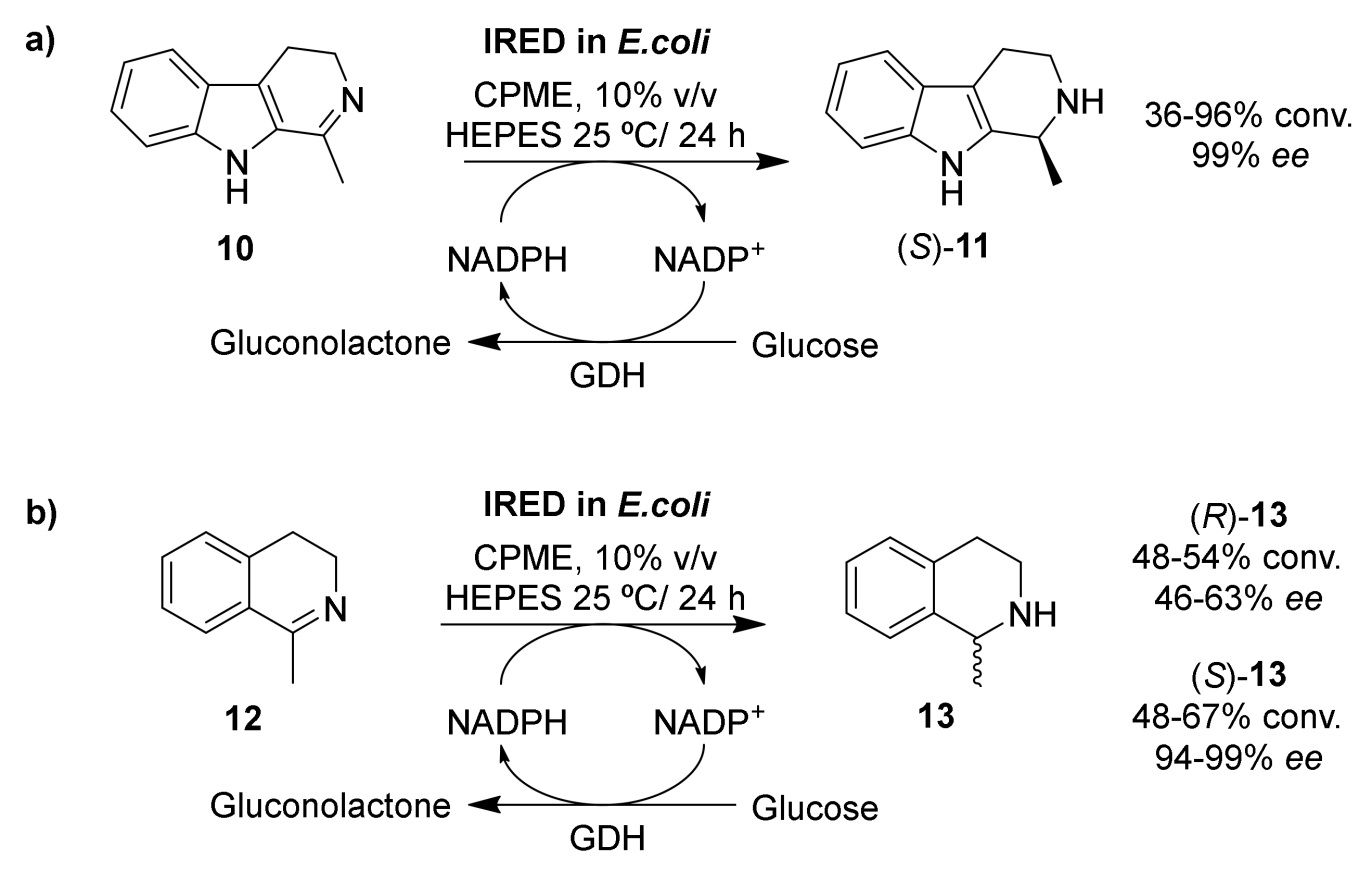
Micro-aqueous systems in CPME using *E. coli* cells expressing IREDs for the asymmetric reduction of: **a**) β-carboline; and **b**) isoquinoline.

In 2015, CPME was employed as cosolvent in the reduction of a set of activated alkenes catalyzed by different enoate reductases [53]. These flavin-dependent enzymes are able to catalyze the asymmetric hydrogenation of C=C double bonds, yielding the corresponding chiral reduced compounds [54,55] and requiring nicotinamides as cofactors in order to perform their activity. Unfortunately, poor conversions (<10%) were achieved when using CPME at 20% *v*/*v* during the optimization of reaction conditions for the reduction of six compounds, where the enzyme did not show a sufficient stability towards solvent such as 1-butanol and ethers. Successful examples of the use of CPME as solvent in bioredox processes have been reported for the selective synthesis of chiral valuable amines by the novel imine reductases (IREDs). IREDs are NADPH-dependent oxidoreductases that catalyze the asymmetric reduction of imines and iminium ions to the corresponding chiral secondary and tertiary amines, employed in biocatalysis since 2010 [56,57]. *E. coli* cells expressing IREDs have been employed in micro-aqueous systems for the selective reduction of β-carboline harmane (**10**) and 1-methyl-3,4-dihydroisoquinoline (**12**, Scheme 5) [58]. The biocatalysts only showed activity in methyl *iso*-butyl ketone and CPME among the organic solvents tested and the buffer concentration was optimized to 10% *v*/*v*. Overall results showed that most of the screened IREDs achieved moderate (>46%) to excellent (>94%) enantioselectivities in the synthesis of (*S*)-**11** and both enantiomers (*R*)- and (*S*)-**13**. This system opens the possibility to expand the substrate scope of IREDs towards more hydrophobic molecules.

**Scheme 6:**
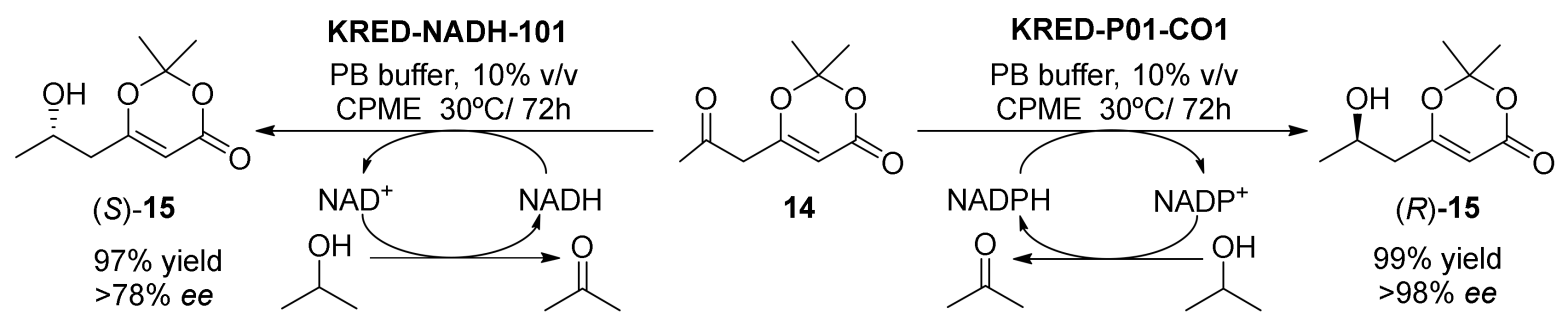
Enantioselective reduction of β-ketodioxinones by ADHs to both enantiomers of β-hydroxydioxinones using isopropanol as sacrificial electron donor.

Moreover, CPME has also been applied as cosolvent in ADH-catalyzed reductions, where a higher yield and stereocontrol in the reactions were achieved compared to their traditional synthetic alternatives. One of these examples is the synthesis of the building block molecule β-hydroxydioxinone **15** by the ADH-catalyzed enantioselective reduction of β-ketodioxinone **14** (Scheme 6) [59]. Interestingly, the use of ethereal solvents such as THF, 2-MeTHF, CPME and MTBE in 5–25% *v*/*v* proportions, increased the yield of the process from 68% in buffer phosphate pH 7 to the highest 98% achieved with 10% CPME. In addition, using this cosolvent the catalyst loaded could be reduced from 5% weight to 1%, while maintaining the same high yields. Both (*R*)- and (*S*)-enantiomers where accessible to a large scale (20 g) with high 97–99% yield and stereoselectivities using stereocomplementary commercial engineered ADHs from the Codexis Ketoreductase Screening Kit in 10% *v*/*v* CPME. In addition, the system could be expanded to the synthesis of more than 20 other β-hydroxydioxinones with overall excellent yields (>90%) and enantioselectivities (>90% *ee*), widening the substrate scope and giving access to new molecules whose synthesis was not possible before in a stereoselective manner.

**Scheme 7:**
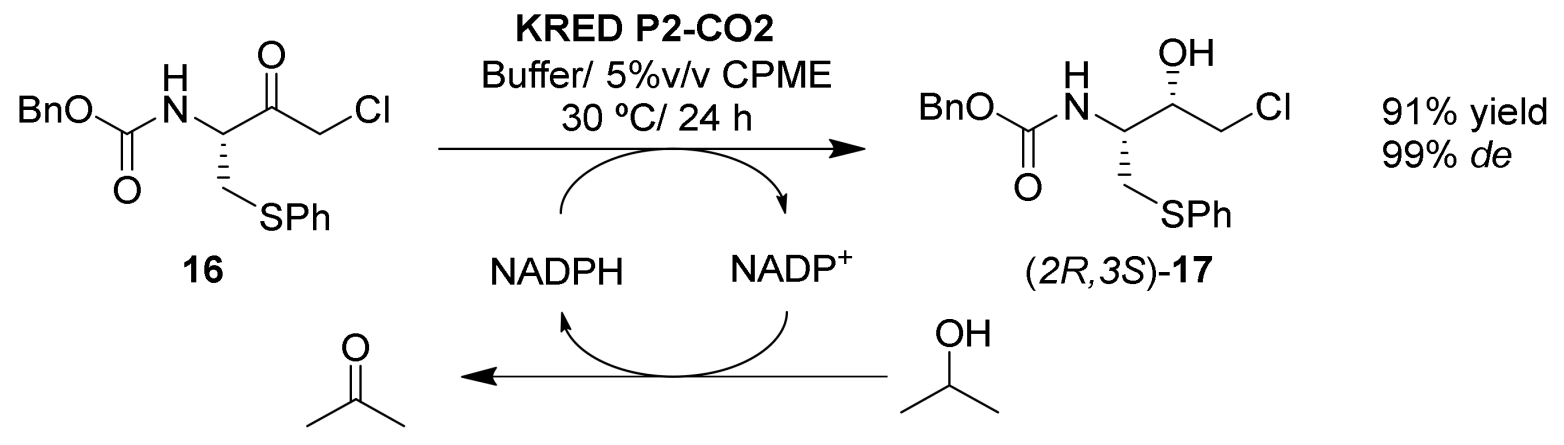
Stereoselective bioreduction of α-chloroketone to (2*R,*3*S*)-chlorohydrin **17** by KREDs in the presence of 5% CPME as cosolvent. Cofactor recycling was performed using the isopropanol-acetone oxidative cycle.

CPME has also been selected as cosolvent due to the higher yield achieved in the bioreduction step of the chemoenzymatic synthesis towards the anti-HIV drug Nelfinavir [60]. The process consisted on the chemical preparation of an α-chloroketone **16** and its subsequent stereoselective reduction catalyzed by ADHs to (2*R,*3*S*)-chlorohydrin **17** (Scheme 7), which is a key-intermediate in the synthesis of Nelfinavir. Among the commercial enzymes tested, two showed the desired stereoselectivity towards the enantiomer of interest and the addition of 5% *v*/*v* CPME as cosolvent maximized both conversion (99%) and stereoselectivity (99% *de*). The higher yield and stereocontrol of this biocatalytic method is the main advantage over the classical reduction methodologies.

**Scheme 8:**
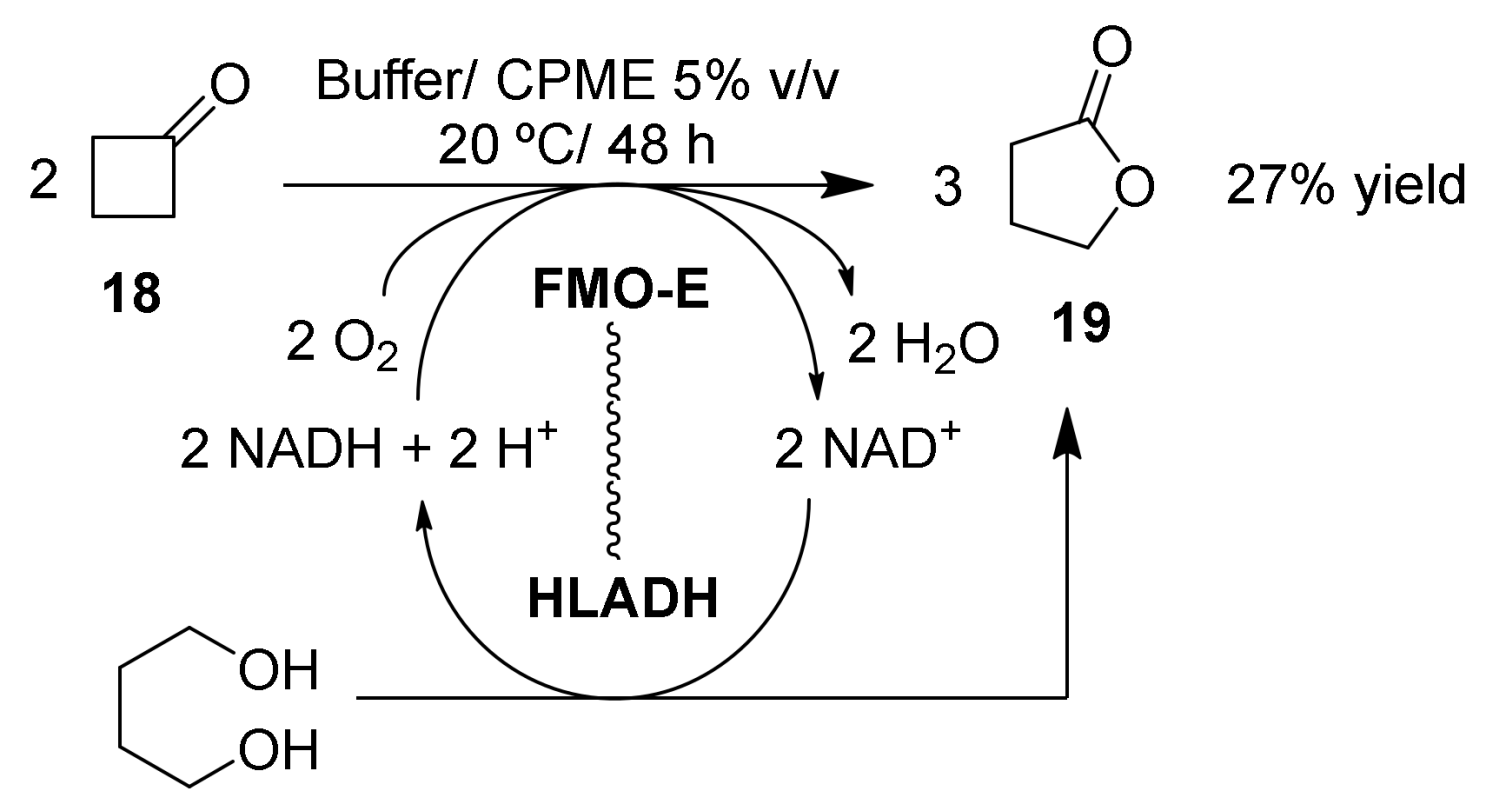
Convergent cascade reaction for the synthesis of γ-butyrolactone by fused FMO-E and HLADH using CPME as cosolvent.

Another example of micro-aqueous system in CPME is the convergent cascade affording γ-butyrolactone **19** as a model lactone product, starting from cyclobutanone (**18**) and butane-1,4-diol (Scheme 8) [61].

Herein, an elegant solution to make enzyme-coupled cofactor regeneration possible in low-water media by fusing two enzymes (monooxygenase FMO-E and alcohol dehydrogenase HLADH) to minimize the “cofactor travel distance” avoiding the nicotinamide cofactor degradation by organic solvents is proposed. A study of the solvent effect showed that the ethereal solvents such as MTBE and CPME were suitable solvents resulting in about 27% yield, while in other lower log P value solvents tested (acetonitrile, isopropyl alcohol and THF) there was not product formation after long reaction times. This is another example in which the higher hydrophilicity of the solvent strongly affected the substrate conversion due to the stripping of the essential enzyme-bound water and decreasing protein flexibility.

In line with CPME, 2-MeTHF has also been proposed as an eco-friendly solvent for different biocatalyzed redox processes, since it usually performs like traditional solvents such as THF but with better characteristics. Besides its attractive physical and chemical properties such as its low miscibility with water, low boiling point and the possible biosynthesis from biomass-derived furfural or levulinic acid (Figure 1), preliminary studies shown also its fast (bio)degradation and low toxicity and it has already been approved for its use in the pharmaceutical industry [18,62].

**Scheme 9:**
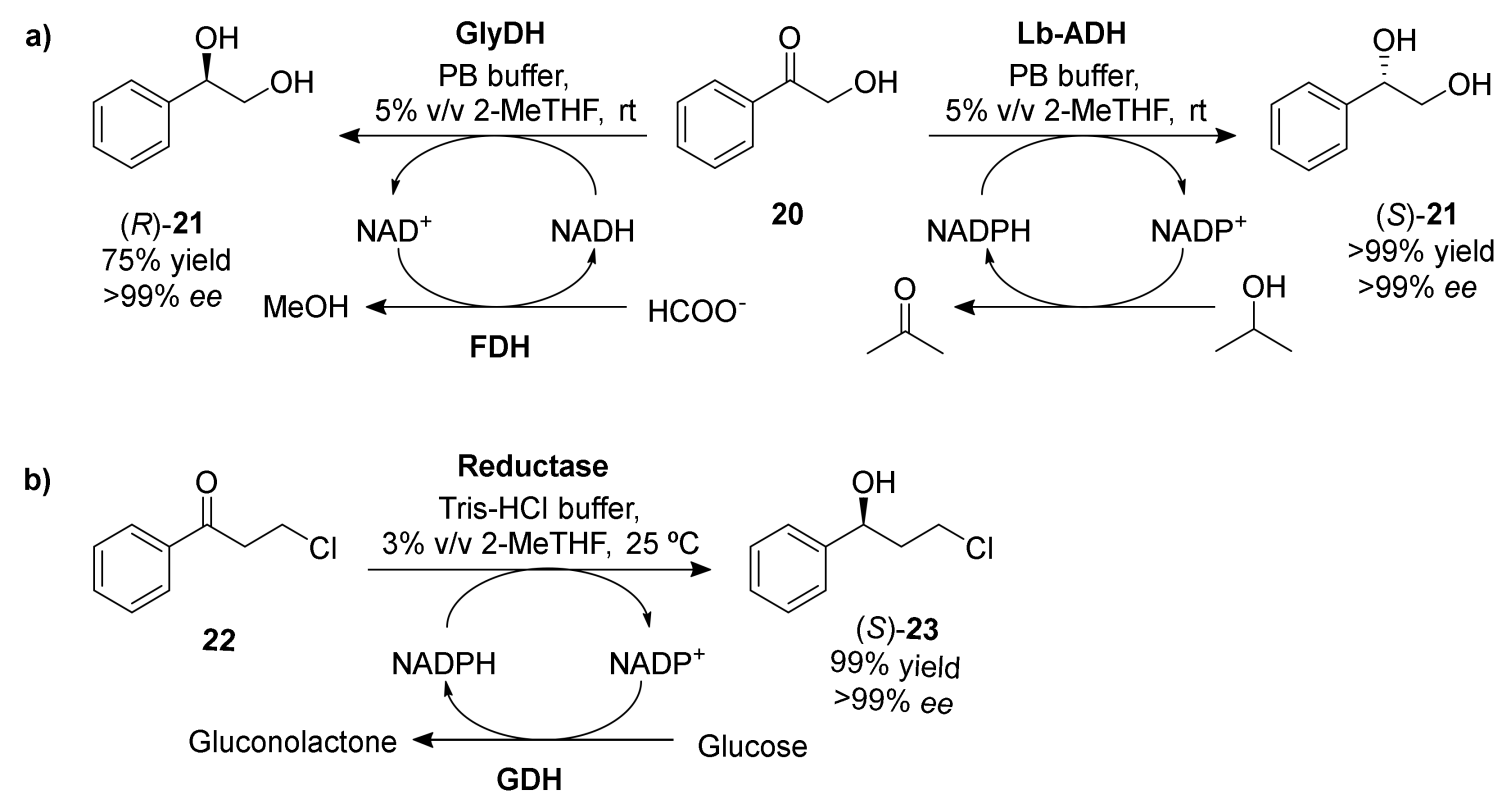
Biocatalyzed enantioselective reductions of **a**) 2-hydroxy-1-phenyl-1-ethanone (**20**), and **b**) 2-chloro-1-phenyl-1-propanone (**22**) in presence of 3–5% (*v*/*v*) 2-MeTHF as cosolvent.

The use of 5% *v*/*v* of 2-MeTHF in phosphate buffer pH 8 was demonstrated to be an efficient alternative to DMSO and MTBE in the lyase-catalyzed reaction towards chiral α-hydroxy ketones, leading to a diminished *E* factor in the process [63]. This biocatalyzed reaction was later combined in a one-pot cascade procedure with ADHs and glycerol dehydrogenase (GlyDH) for the synthesis of α-aryl vicinal diols (Scheme 9a) [64]. After optimization, high yields (75–99%) and excellent enantioselectivity (>99% *ee*) for both the (*R*)- and (*S*)-**21** were achieved in the bioreduction of the α-aryl-2-oxo-1-hydroxy ketone **20**, using LbADH and the glycerol dehydrogenase from *Cellulomonas sp.* (GlyDH), respectively. In addition, the methodology was extended to more challenging aromatic aldehydes like furfural, for the synthesis of chiral furan-1,2-diols again with high yield (87%) and enantioselectivity (>99% *ee*). The suitability of using 2-MeTHF as cosolvent (3% *v*/*v*) in whole-cell biotransformations was also demonstrated in the conversion of 3-chloro-1-phenyl-1-propanone **22** to (*S*)-3-chloro-1-phenylpropanol **23** [65], employing recombinant *E. coli* cells containing the *Saccharomyces cerevisiae* YOL151W reductase and glucose dehydrogenase for the NADPH cofactor regeneration in Tris-HCl buffer (pH 7.5) (Scheme 9b). The system showed high efficiency with a 99% yield and complete enantioselectivity for the formation of (*S*)-**23**.

Table 1 summarized the biocatalysts employed in presence of CPME and 2-MeTHF for bioreduction and biooxidation processes.

## 4. Deep-Eutectic Solvents in Bioreductions/Biooxidations

**Figure 2:**
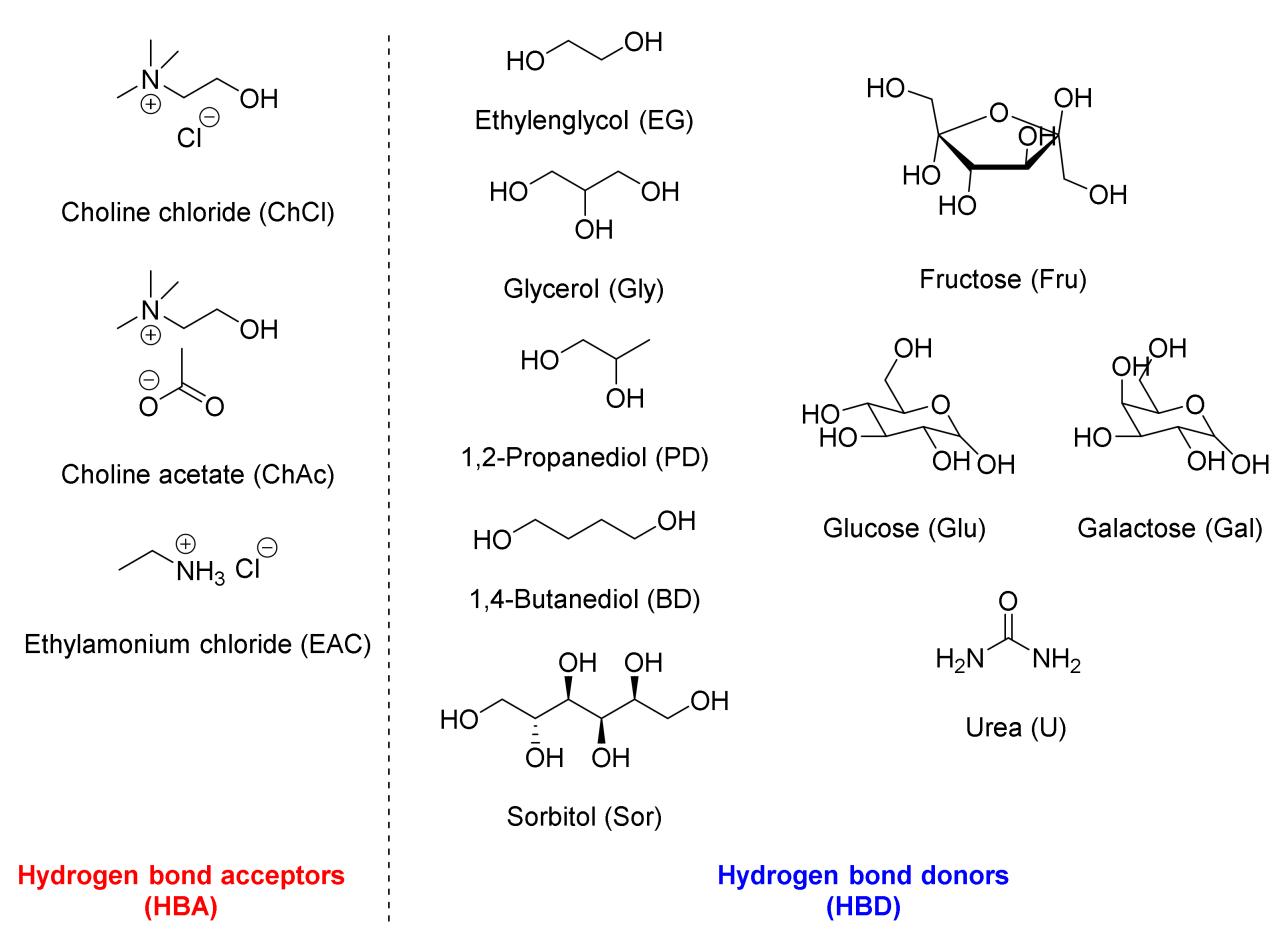
Structure of the components of the (NA)DESs described in the present revision.

Deep eutectic solvents (DESs) are liquid compounds obtained by mixing a hydrogen bond donor (HBD) with a hydrogen bond acceptor (HBA) at a determined molar ratio at high temperature (50–80 °C). After stirring for some hours a liquid is formed, which maintains this state even after cooling down at room temperature. The hydrogen bonds formed reduced the overall melting point, forming the eutectic mixture which can be employed without further purification, representing an advantage as the synthesis of DESs is a waste-free procedure. HBAs more typically employed are quaternary ammonium salts, as choline or ethylamonium salts, whereas different types of hydrogen bond donors can be employed in the preparation of these liquids (Figure 2), typically including polyols as ethylenglycol (EG), glycerol (Gly), xylitol or sorbitol (Sor); organic acids such as oxalic, malic, levulinic or malonic acid; sugars as glucose (Glu), fructose (Fru) or galactose (Gal), and other compounds such as urea (U) or acetamide. When the DESs are formed by natural components, including sugars, aminoacids or polyols, they can be also called Natural Deep Eutectic Solvents (NADESs).

Apart from their easy preparation, DESs present a wide set of advantages, including a high biodegradability, non-volatility, non-flammability and low toxicity [66,67]. By these reasons, DESs have constituted a greener alternative as (co)solvents to develop asymmetric catalytic transformations. In addition, it is possible to modify the structure of these solvents in order to tune their physico-chemical properties, thus being in principle possible to design DESs for specific applications.

Seminal work in the field of biocatalyzed reactions in presence of DESs was developed by Kazlauskas et al. in 2008 [68], employing a set of choline chloride-based solvents in different processes catalyzed by hydrolases. Since them, DESs have been widely employed as solvents or cosolvents in enzymatic catalysis [69,70,71]. As usual, most of the examples of biocatalyzed reactions involving DESs have been catalyzed by hydrolases, as this type of enzymes are able to carry out their activity in several non-aqueous media. But the application of DESs is not only restricted to hydrolysis or synthetic processes, being described some other examples with dehalogenases [72], lyases [73] and also with oxidoreductases [74,75].

Apart from the inherent positive effect of employing DESs as (co)solvent in catalytic processes, due to their environmentally friendly properties, these compounds usually can present a further benefit on the biocatalyzed reactions, by increasing the activity and/or the selectivity of the processes. In this revision, some recent advances of the application of (NA)DESs in biocatalysis will be shown, both in reductive and in oxidative processes.

### 4.1. Bioreductions in Presence of (NA)DESs

In the last years, several examples of biotransformations catalyzed by ADHs in presence of (NA)DESs have been developed. Whole-cells systems have been employed in most of the examples described, whereas only very recent process has been developed using the catalysts as isolated enzymes.

**Scheme 10:**
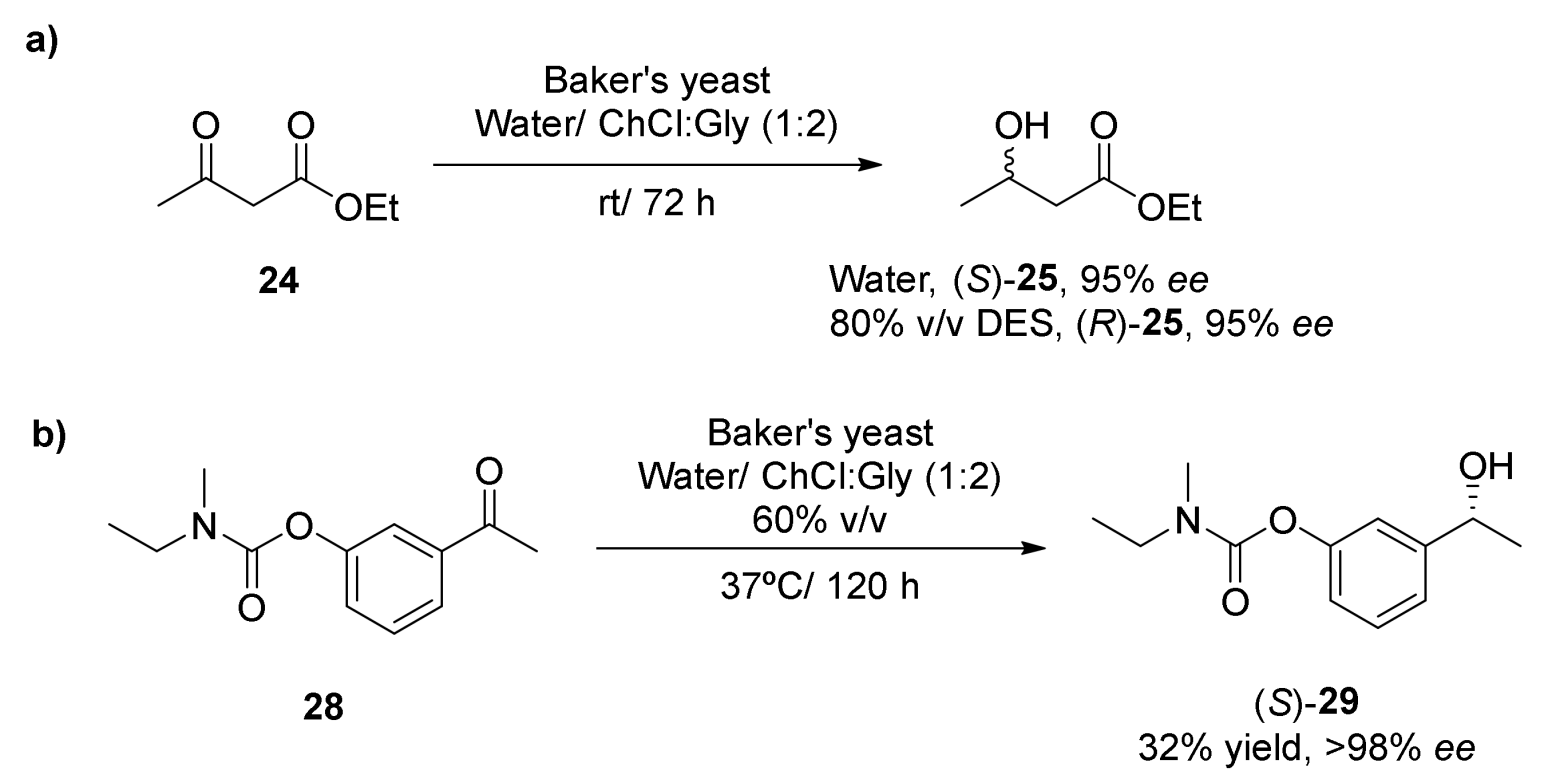
Biocatalyzed reductions using ChCl:Gly (1:2) as cosolvent employing baker’s yeast of **a**) ethyl acetoacetate (**24**), and **b**) rivastigmine precursor **28**.

One of the first approaches in the application of DESs in ADH-catalyzed bioreductions was developed in 2014 by Domínguez de María et al., employing choline chloride-glycerol ChCl:Gly (1:2)-buffer systems in the bioreduction of ethyl acetoacetate (**24**) catalyzed by baker’s yeast (Scheme 10a) [76]. The addition of different amounts of the DES in buffer led to lower conversions at 72 h than when performing the bioreduction in pure water, but it was observed that for DES contents between 50 and 90% *v*/*v*, the baker’s yeast cells were still active in the bioreduction after 200 h, indicating that the cells maintained their integrity after that time and the intracellular oxidoreductases were still active. The selectivity of the process was also analyzed, being observed the interesting result than depending on the amount of ChCl:Gly (1:2) employed, a different enantiomer of the final alcohol **25** can be obtained. In pure water, the baker’s yeast whole cells led to the formation of the (*S*)-enantiomer with 95% *ee*. Opposite, working in DESs systems with up to 20% *v*/*v* water, high (*R*)-enantioselectivity was obtained. This result can be explained by the presence of several enzymes with redox activity in the baker’s yeast genome. In a further development on the application of baker’s yeast in presence of different DESs [77], phenylacetone (**26**) was selectively reduced to 1-phenylpropan-2-ol (**27**), as shown in Table 2. When the process was performed in water, the (*S*)-alcohol was recovered with 88% yield and 96% *ee*, whereas no reaction was observed in neat DESs, being required at least some amount of water to observe enzymatic activity. The reactions using 90–80% *v*/*v* of the DESs choline chloride/D-fructose, [ChCl:Fru (3:2 weight/weight)] or ChCl:Gly (1:2) afforded 1-phenylpropan-2-ol with lower yields than in water, but in this case it was possible to obtain the (*R*)-enantiomer with moderate to good selectivity, showing again a reversal in the system enantiopreference by modifying the reaction medium.

Thus, both enantiomers of the desired product can be achieved using very cheap and environmentally friendly catalysts and reaction media. Authors explain these results by the inhibition of the (*S*)-ADHs at high DESs contents, allowing the formation of the (*R*)-alcohols in these conditions. Baker’s yeast-catalyzed bioreductions were extended to other prochiral ketones, following the same trend for compounds with a space between the carbonyl moiety and the aromatic ring of two carbon bonds: High conversions and (*S*)-selectivity in pure water and lower yields and (*R*)-alcohols in 80 or 90% *v*/*v* ChCl:Gly (1:2). The use of ketones with a longer space does not lead to the switch in the enantiopreference. Baker’s yeast resting cells have been also tested in the reduction of a rivastigmine precursor (**28**) to the corresponding chiral alcohol **29** in presence of different amounts of ChCl:Gly (1:2), as shown in Scheme 10b [78]. The addition of this DES in 60% *v*/*v* led to a slight increase in the product yield respecting in water alone, whereas a 98% *ee* of the (*S*)-enantiomer was measured. In this process, no stereoinversion was observed when employing the DES.

Apart from using baker’s yeast as biocatalyst for the reduction of prochiral ketones, several ADHs have been employed in the biosynthesis of optically active alcohols in presence of different amounts of NADES. 

**Scheme 11:**
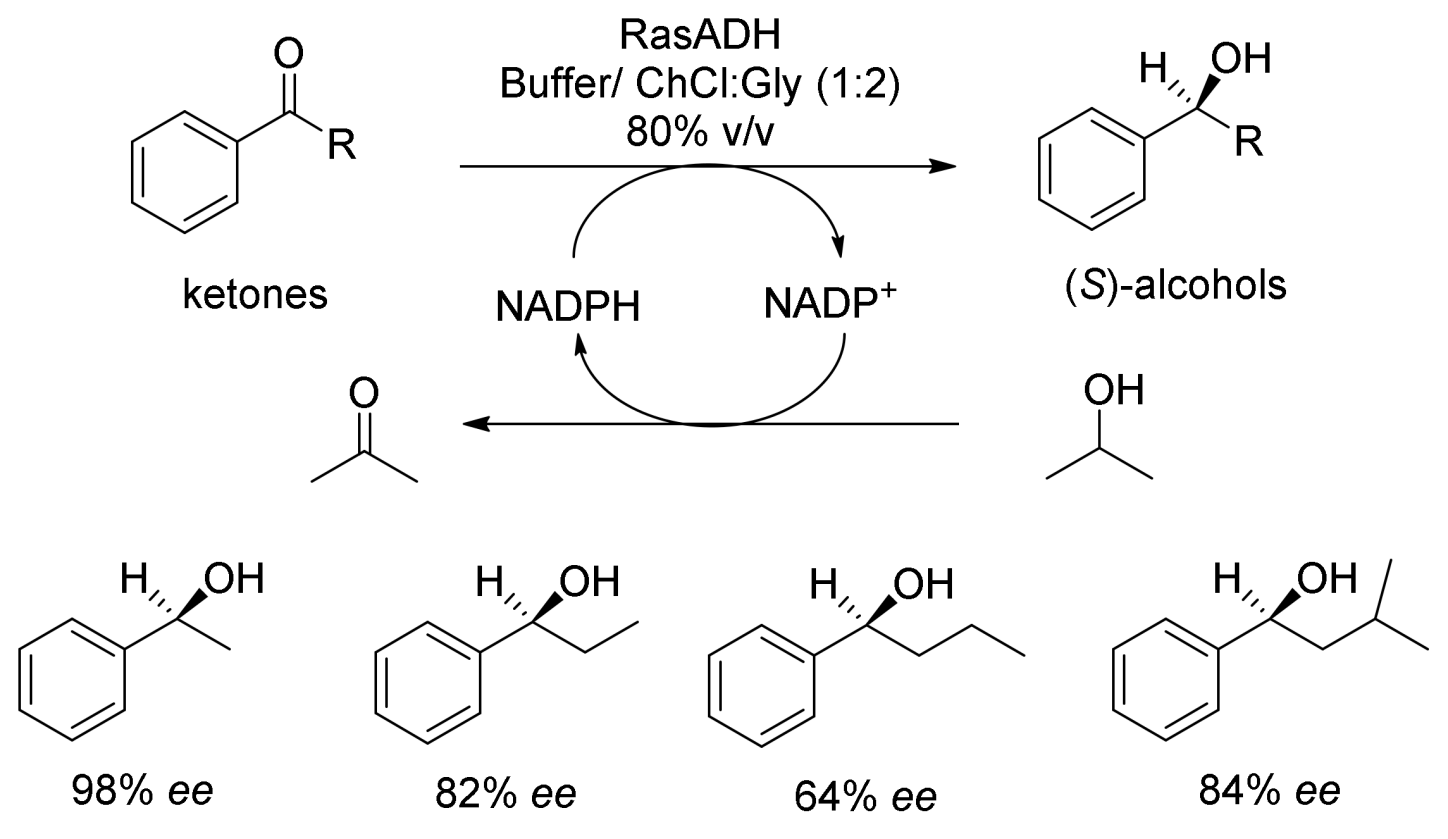
RasADH-catalyzed reduction of aromatic ketones using ChCl:Gly (1:2) as cosolvent to yield chiral aromatic *sec*-alcohols.

In 2015, the stereoselective reduction of prochiral ketones biocatalyzed by different ADHs overexpressed in *E. coli* was carried out [79]. Initial experiments showed a high performance when working with *E. coli* cells expressing the ADH from *Ralstonia* sp. (RasADH), as this biocatalyst remained with complete activity at 60–70% *v*/*v* of ChCl:Gly (1:2) and even present some activity at 95% *v*/*v* of the DES. The conversions obtained in the bioreduction depended on the ancillary co-substrate employed to regenerate the nicotinamide cofactor, being observed much higher conversions when applying 1-propanol or 2-propanol than ethanol. A significant result was observed in the bioreductions stereoselectivity, as the chiral secondary alcohols were obtained with higher optical purities when presenting high contents of DES (80% *v*/*v*) than in buffer alone, especially when testing bulky ketones (Scheme 11). Biotransformations were also studied with other choline based-DES, including ChCl:U (1:2) and ChCl:EG (1:2). For both DESs, the selectivity improvement respecting buffer alone was maintained, but lower conversions were achieved respecting the glycine-based DES.

The same positive effect on the selectivity in the bioreduction of ketones by using DESs as cosolvents was also observed when reducing 2-octanone to (*R*)-2-octanol by *Acetobacter pasteurianus* GIM1.158 cells [80]. When employing a 10% *v*/*v* ChCl:EG (1:2), the optimal substrate concentration was 60 mM, three times higher than in aqueous system alone, indicating that the DES can act as good substrate reservoir. Under these optimal conditions, (*R*)-2-octanol can be obtained with 95.7% yield and 98.9% optical purity. The authors analyzed the effect of ChCl:EG (1:2) on the biocatalyst, being observed a good biocompability of the DES with the cells and an increase in the cell membrane permeability in order to explain the positive effect of this solvent in the reaction efficiency.

**Scheme 12:**
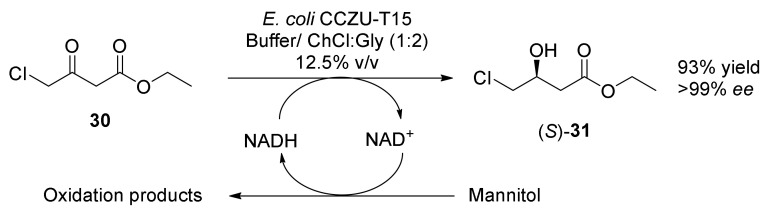
Synthesis of ethyl (*S*) 4-chloro-3-hydroxybutanoate in 12.5% *v*/*v* ChCl:Gly (1:2) catalyzed by *E. coli* CCZu-T15 cells.

Several DESs at 10% *v*/*v* were employed as cosolvents in the bioreduction of ethyl 4-chloro-3-oxobtunoate (**30**) into ethyl (*S*)-4-chloro-3-hydroxybutanoate (**31**), a precursor of cholesterol-lowering hydroxymethylglutaryl-CoA reductase inhibitors, catalyzed by *E. coli* CCZU-T15 cells (Scheme 12) [81].

The different DESs studied do not affect to the system selectivity (≥99% *ee* in all cases), being achieved improvements in the activity by 1.08, 1.14 and 1.21-fold respecting buffer alone when using ChCl:U (1:1), ChCl:EG (1:2) and ChCl:Gly (1:2). Further optimization of the system led to a better performance in the preparation of (*S*)-**31** with 12.5% *v*/*v* of ChCl:Gly (1:2) in buffer pH 6.5 when starting from 200 mM of prochiral ketone. In these conditions, (*S*)-alcohol was recovered with excellent yields.

**Scheme 13:**
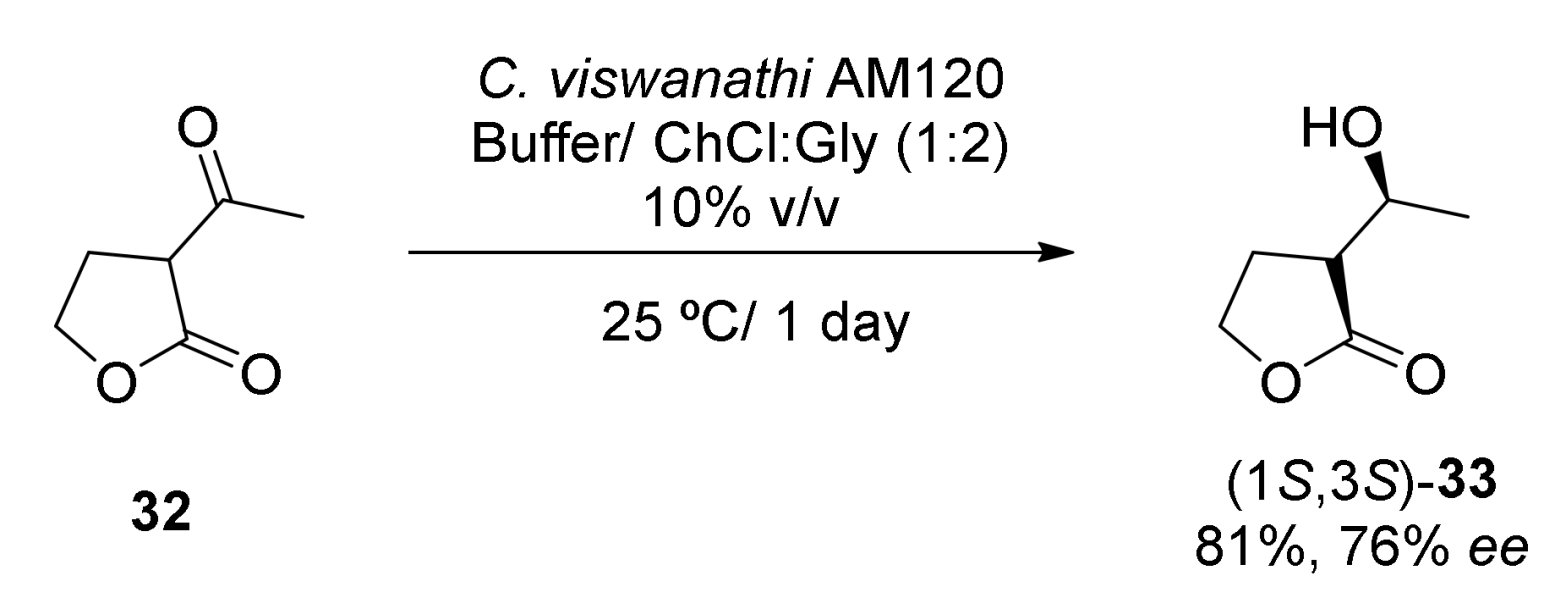
Preparation of lactone (1*S*,3*S*)-**33** catalyzed by *Candida viswanathi* AM120 cells in buffer containing 10% *v*/*v* ChCl:Gly (1:2).

The synthesis of optically pure α’-1-hydroxyethyl-γ-butyrolactone (**33**), valuable synthons in organic chemistry, has been recently developed by Maçzka et al. by performing the reduction of α-acetylbutyrolactone (**32**) catalyzed by different yeasts (Scheme 13) [82]. Some *Yarrowia* strains as well as *Candida viswanathi* AM120 led to very good results, being obtained the *anti* diastereoisomers with high selectivity. This process was optimized by testing several reaction parameters. Thus, the addition of ChCl:Gly (1:2) conduced to more efficient processes when employing *Candida viswanathi* AM120, as the bioreductions in phosphate buffer pH 7.0 in 10 or 25% *v*/*v* of this DES were faster than in buffer alone. A slight increase in the process selectivity was achieved, being recovered as major product the *anti*-(1*S*,3*S*)-enantiomer of **33** with 76% *ee* and almost complete diastereoselectivity.

The bioreduction of 2-hydroxyacetophenone (**20**) to the corresponding (*R*)-1-phenyl-1,2-ethanodiol (**21**) catalyzed by cells of *Kurthia gibsonii* SC0312 was studied using five different DESs as cosolvents [83]. The use of choline chloride/1,4-butanediol, ChCl:BD (1:4) at 2% *v*/*v*, led to an excellent biocompatibility, being observed an increase in the catalytic rate of the reduction by 22% respecting the use of buffer pH 7.5 alone. After optimizing all the reaction parameters, an excellent process could be afforded by reducing 80 mM of 2-hydroxyacetophenone in the presence of *K. gibsonii* SC0312 cells in buffer containing 2% *v*/*v* ChCl:Bd (1:4). Thus, enantiopure (*R*)-1-phenyl-1,2-ethanediol was obtained in 80% yield at 30 mg/mL wet cells.

**Scheme 14:**
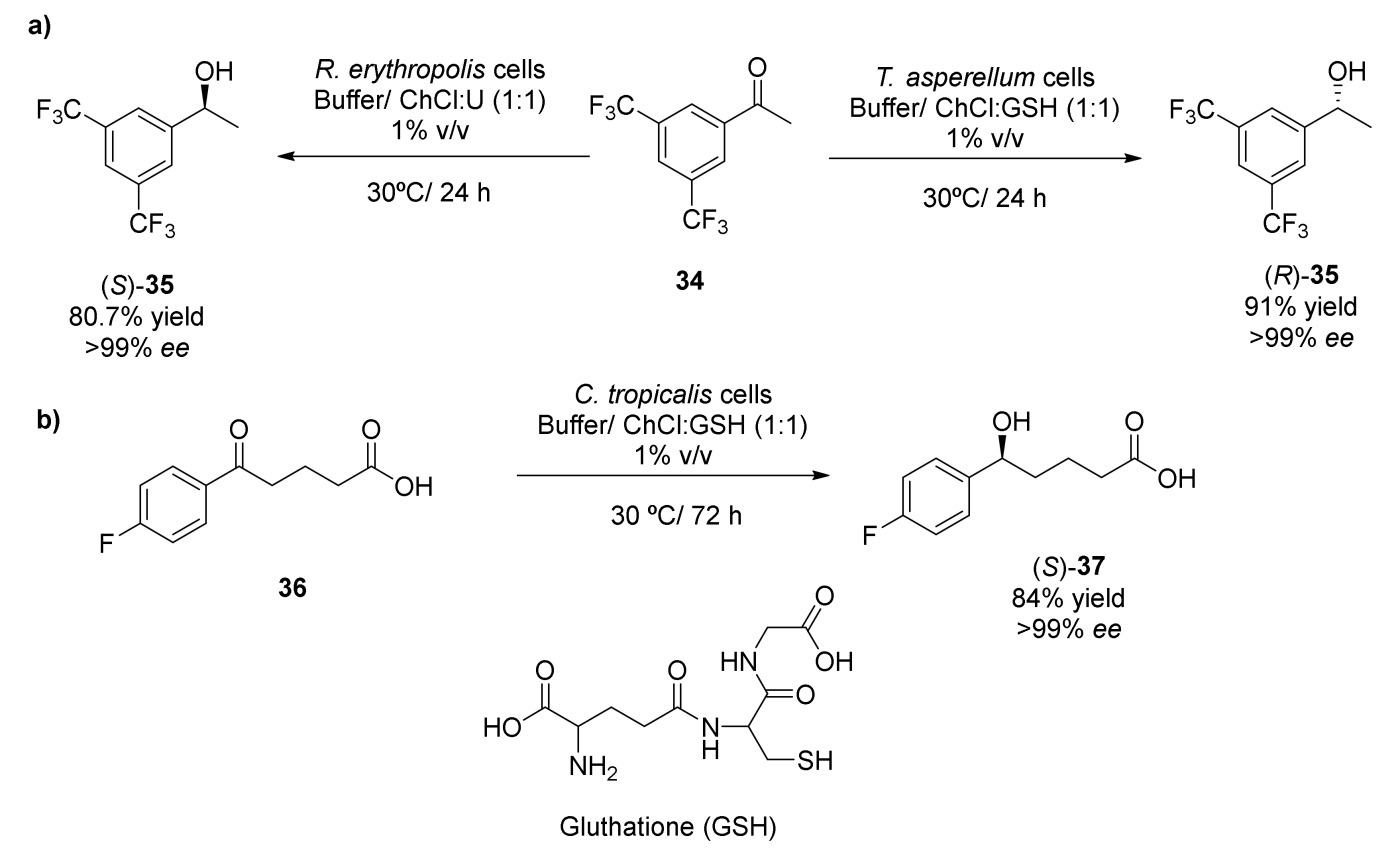
Choline-based DESs employed in the desymmetrization of: **a**) prochiral 3,5-bis(trifluoromethyl) acetophenone (**34**), and **b**) 5-(4-fluorophenyl)-5-oxopentanoic acid (**36**) catalyzed by different whole-cells containing ADHs.

In 2020, the selective bioreduction of 3,5-bis(trifluoromethyl)acetophenone (**34**) to (*S*)-3,5-bistrifluoromethylphenyl ethanol (**35**), a valuable intermediate in the synthesis of the NK-1 receptor antagonist, was performed using *Rhodococcus erythropolis* XS1012 cells (Scheme 14.a, left) [84]. Due to the low yields obtained in phosphate buffer, a set of choline chloride-based DESs were tested as cosolvents in the bioreductions in 1% *w*/*v*. Among all the DESs tested, the best results were achieved employing ChCl:U (1:1), being possible to obtain a much higher conversion than using buffer alone. As stated for other authors, it is hypothesized a positive effect of the DES in the cells membrane permeability. At the optimal conditions, 300 mM of **34** were reduced by the cells in phosphate buffer pH 6.5 containing 1% *w*/*v* ChCl:U (1:1), yielding after 24 h at 30 °C a 80.7% yield of enantiopure (*S*)-alcohol.

Bioreduction of ketone **34** has been also tested employing *Trichoderma asperellum* ZJPH0810 cells in buffer containing different (NA)DESs (Scheme 14a, right) [85]. For this biotransformation the oligopeptide gluthatione (GSH), a tripeptide formed by glutamine, glycine and cysteine, was employed as HBD in order to form a DES with choline chloride in equimolar proportion. When this novel DES was employed in the preparation of (*R*)-**35**, it was possible to obtain the enantiopure alcohol with 91% yield after 24 h, much higher than the achieved in buffer alone (53%). Authors established than using 1% *v*/*v* of ChCl/GSH (1:1), GSH is able to form hydrogen bonds with ChCl and with carbonyl group’s oxygen in the ketone, thus its electrophilicity, facilitating the proton acceptance from NADH and promoting coenzyme regeneration. ChCl:GSH (1:1) was also analyzed in other whole-cells biocatalyzed reductions. Thus, this cosolvent can be employed in the bioreduction of 100 mM of ketone **34** to (*S*)-**35** catalyzed by *Candida tropicalis* 104 cells, achieving an 87.6% yield of the enantiopure alcohol. 5-(4-Fluorophenyl)-5-oxopentanoic acid (**36**) was selectively reduced to (*S*)-(4-fluorophenyl)-5-hydroxypentanoic acid (**37**), a valuable synthon in the synthesis of the cholesterol-lowering drug Ezetimibe, by *Candida parapsilosis* ZJPH1305 cells in 1% *v*/*v* ChCl:GSH (1:1) (Scheme 14b). The enantiopure alcohol was obtained with a much higher yield using DES as cosolvent than in aqueous buffer alone, 84% vs. 66% yield, respectively, after 72 h at 30 °C.

Carrot roots have been employed as biocatalysts for the bioreduction of 1-(3,4-dimethyl-phenyl)ethanone in water containing five choline-based NADESs [86]. The addition of these cosolvents (20–70% *v*/*v*) in the reaction medium at different concentrations led to much lower conversion than in pure water. The type of hydrogen bond donor of the DES has a huge effect on the enzymatic activity, with the highest conversion obtained for glucose > xylose > glycerol > xylitol > ethylene glycol. This result can be partially explained by the existence of auxiliary substrates (like sugars) in the medium, which can collaborate in the cofactor-recycling system, thus enhancing the conversions. Increasing the water content has a positive effect in the system for all the DESs tested, being recovered the final alcohol with higher yields. Again, as observed for the baker’s yeast experiments in DESs, the occurrence of high amounts of NADESs led to a change in the enzymatic system enantiopreference: For reactions carried out in water, high optical purity of the (*S*)-alcohol was observed, whereas at high DES contents (50–70%), the (*R*)-alcohol was recovered with good enantiomeric excesses. It was observed that this effect was dependent on the pH of the NADES. When the pH of the reaction media was in the range of 4.0–6.0, the (*R*)-configuration for the alcohol was predominant, while for outer values, (*S*)-enantiomer was preferred.

**Scheme 15:**
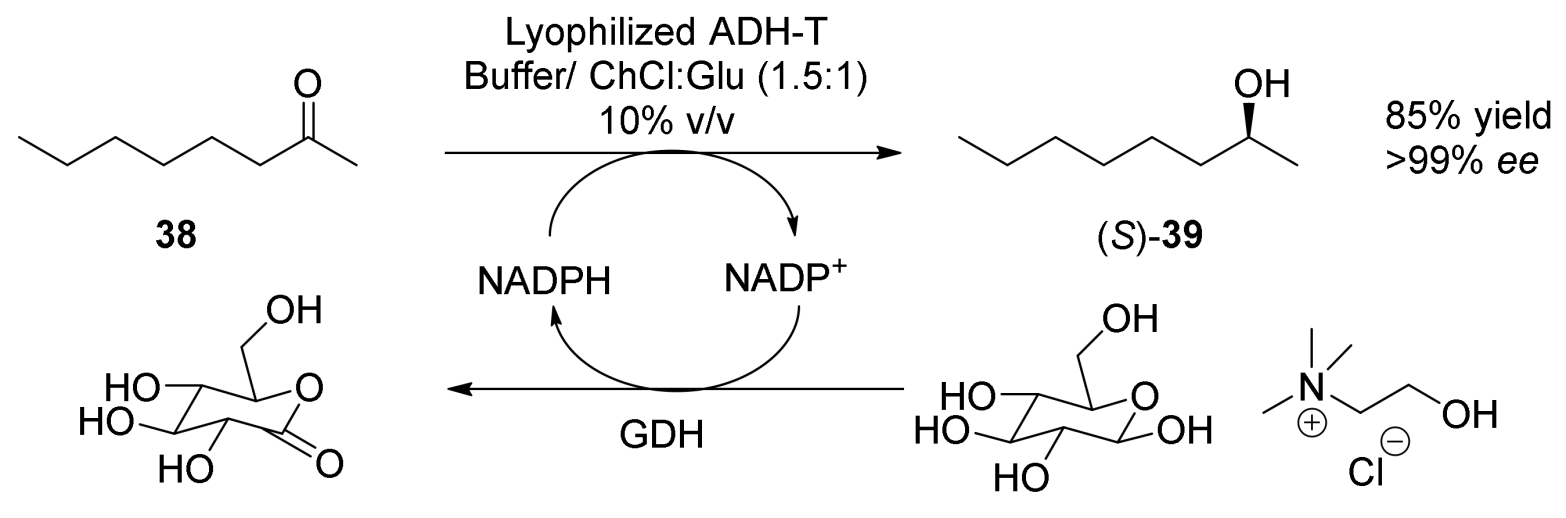
Use of ChCl:Glu (1.5:1) as cosolvent and cofactor regeneration system in the bioreduction of 2-octanone catalyzed by lyophilized cells of ADH-T.

In line with the findings about the effect of HBDs in the cofactor regeneration, a further development in the application of DES with ADHs has been shown in 2019 [87]. Thus, the glucose-based NADES ChCl:Glu (1.5:1) was employed with two purposes in the bioreduction of acetophenone, 2-octanone (**38**) and propiophenone: (1) Cosolvent, and (2) Part of the nicotinamide cofactor recycling system by employing glucose as substrate for this recycling, as shown in Scheme 15. Commercially available GDH-105 from Codexis was selected for the cofactor recycling whereas the NADPH-dependent ADHs from *Lactobacillus brevis* (LbADH), with (*R*)-selectivity, and from *Thermoanaerober ethanolicus* (TesADH), *Thermoanaerober* sp. (ADH-T), *Sphingobium yanoikuyae* (SyADH) and RasADH, presenting (*S*)-selectivity, were tested as lyophilized cells in buffer Tris/HCl pH 7.5 containing 10% *v*/*v* of ChCl:Glu (1.5:1). Complete conversions and enantioselectivities were achieved for LbADH, ADH-T and RasADH for the three ketones. TesADH and SyADH afforded the (*S*)-alcohols with high conversions and good enantiomeric excesses. LbADH, RasADH and ADH-T can perform the bioreductions at higher NADES concentrations, being possible to achieve excellent conversions and selectivities for LbADH at even 80% *v*/*v* NADES, whereas the use of RasADH led to a drop in the enzyme selectivity at 70% *v*/*v*. These three ADHs were studied in the ketone bioreduction at substrate concentrations of 100 mM, obtaining higher conversions in buffer with 10% *v*/*v* of the NADES than in buffer alone. Biotransformations were scaled up to semi-preparative scale. The use of LbADH in buffer/10% *v*/*v* NADES led to a 78% yield of (*R*)-1-phenylethanol, whereas an 85% yield of enantiopure (*S*)-2-octanol (**39**, Scheme 15) and 89% of optically pure (*S*)-1-phenyl-1-propanol were obtained in the bioreductions catalyzed by ADH-T in the same reaction medium. The *E* factor for these processes has been determined by the authors, being obtained values of 236 and 286, respectively. These values are still high, but the highest percentage of them came from the organic solvents employed for the reactions work-up, which indicated that these values can be further improved.

**Scheme 16:**
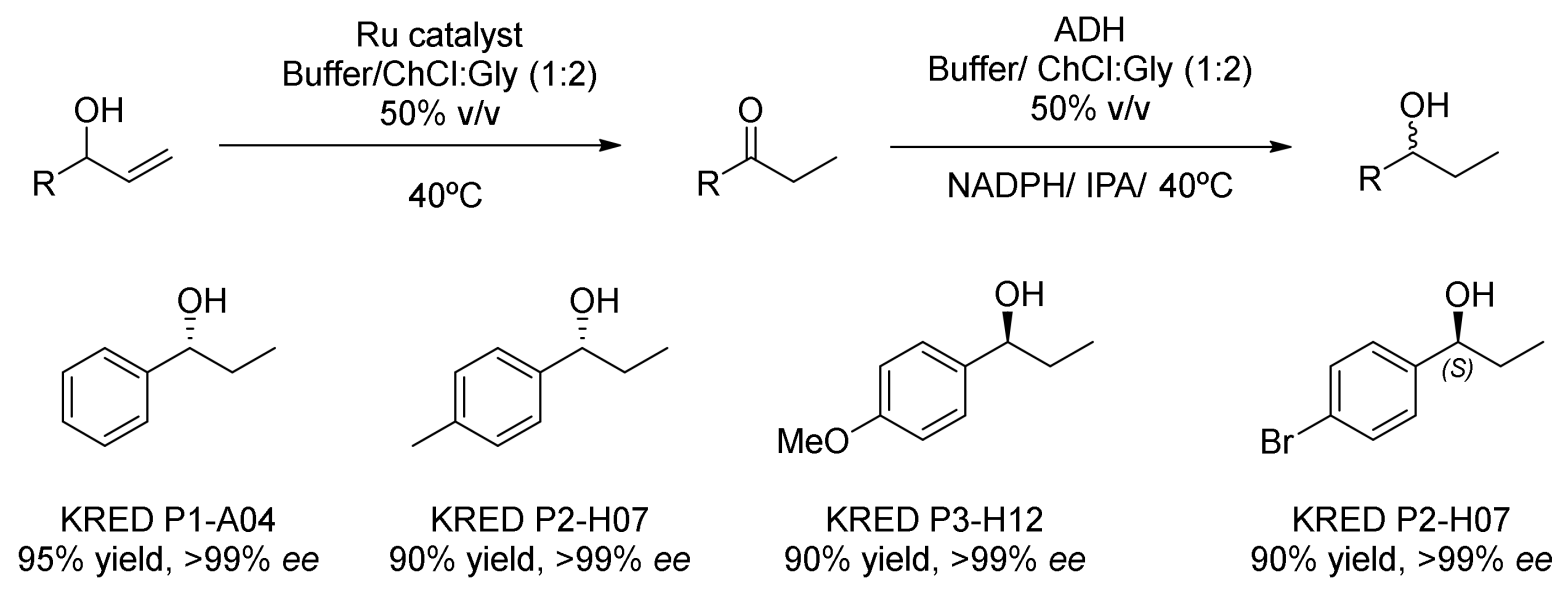
General procedure for the chemoenzymatic cascade to transform allylic alcohols into chiral alcohols by combining a choline chloride-based DES bioreduction with a Ru-catalyzed isomerization.

One of the first examples of the development of biocatalyzed reductions employing isolated ADHs has been shown by Cicco et al. in 2018 in the chemoenzymatic cascade to convert allylic alcohols into chiral alcohols by combining a Ru-catalyzed isomerization of the starting material with the bioreduction of the prochiral ketone catalyzed by different ADHs (Scheme 16) [88]. Both processes were performed in buffer/DESs mixtures.

Regarding the bioreduction step, five different ChCl-based DESs were tested at concentrations from 50 to 80% *v*/*v* in the bioreduction of propiophenone catalyzed by isolated ADHs from Codexis, as well as from isolated *Lactobacillus kefir* ADH (LkADH). Excellent results were obtained using ChCl:Gly (1:2) and choline chloride:sorbitol [ChCl:Sor (1:1)]. The first DES was further employed in the bioreduction of other aromatic ketones, being obtained in general similar results in buffer alone and in buffer with 50% *v*/*v* ChCl:Gly (1:2) regarding the activity and the selectivity.

Higher amounts of the DES led to enzymatic deactivation for most of the biocatalysts employed, but it was possible to achieve excellent results for some of the ketones studied. Enzymes stability was also studied in the presence of the DESs, being observed that although they are slightly less stable than in neat buffer, the isolated ADHs can perform their activity at temperatures up to 40 °C.

The results obtained in the bioreduction of prochiral ketones in presence of ADHs containing some Deep Eutectic Solvents are summarized in Table 3.

### 4.2. Application of DES in Biooxidations

**Scheme 17:**
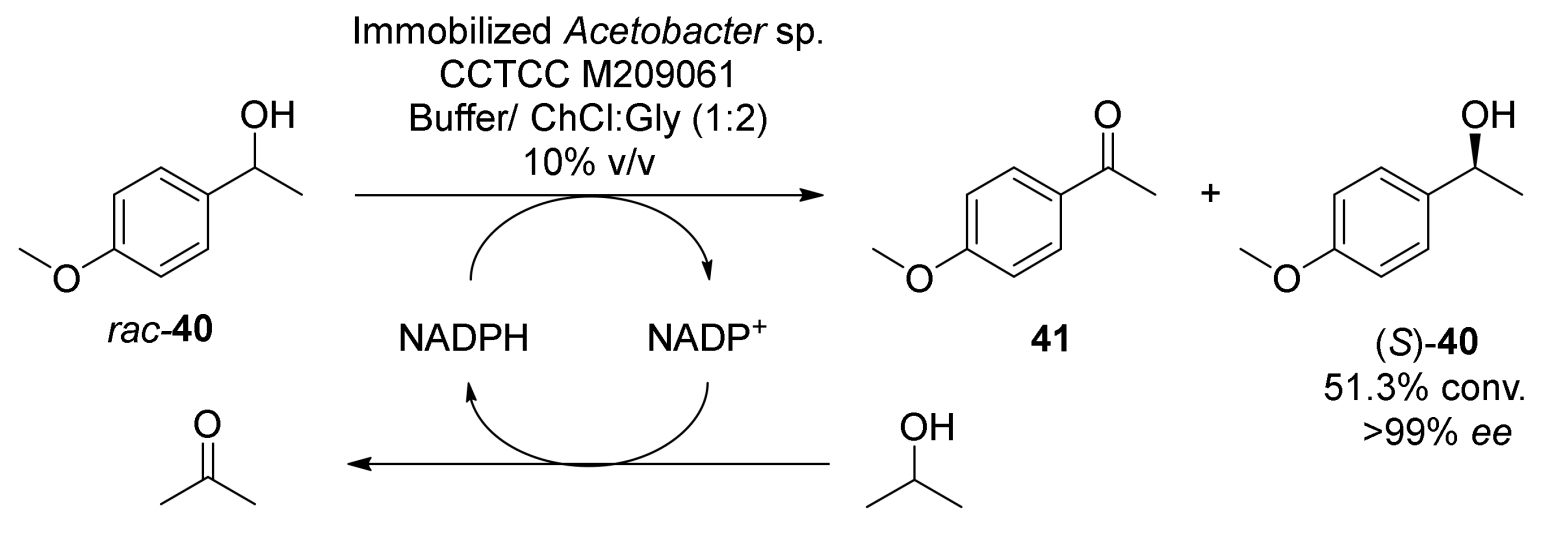
Kinetic resolution of racemic 1-(4-methoxyphenyl)ethanol (**40**) by oxidation catalyzed by immobilized cells of *Acetobacter* sp. CCTCC M209061 in buffer containing 10% *v*/*v* ChCl:Gly (1:2).

As ADHs are able to catalyze the reversible reduction of carbonyl compounds to alcohols, these enzymes have been also employed to synthesize optically active secondary alcohols by performing the selective oxidation of racemic alcohols in kinetic resolution procedures. Thus in 2015, Xu et al. developed the kinetic resolution of 1-(4-methoxyphenyl)ethanol (**40**) catalyzed by *Acetobacter* sp. CCTCC M209061 immobilized cells using as reaction medium buffer and buffer containing different DESs as cosolvents in 20% *v*/*v* (Scheme 17) [89]. After analyzing different choline chloride-based DES, the best performance was found in ChCl:Gly (1:2), being possible to achieve (*S*)-**40** with 98.7% *ee*, in a process with a 49% conversion after 9 h. Further optimization showed the best conditions when using buffer at pH 6.5 containing 10% *v*/*v* of the DES in the biooxidation of 55 mmol/L of **40** carried out at 30 °C. When this process was scaled up on a 500 mL scale, a 51.5% conversion was achieved after 7 h, being recovered 4-methoxyacetophenone (**41**) and enantiopure (*S*)-**40**. Studies on the different DES showed that ChCl:Gly (1:2) showed a slight increase in the membrane permeability of the *Acetobacter* sp. cells, which can partially explain the good results obtained using DESs as cosolvents in this oxidative kinetic resolution.

**Scheme 18:**
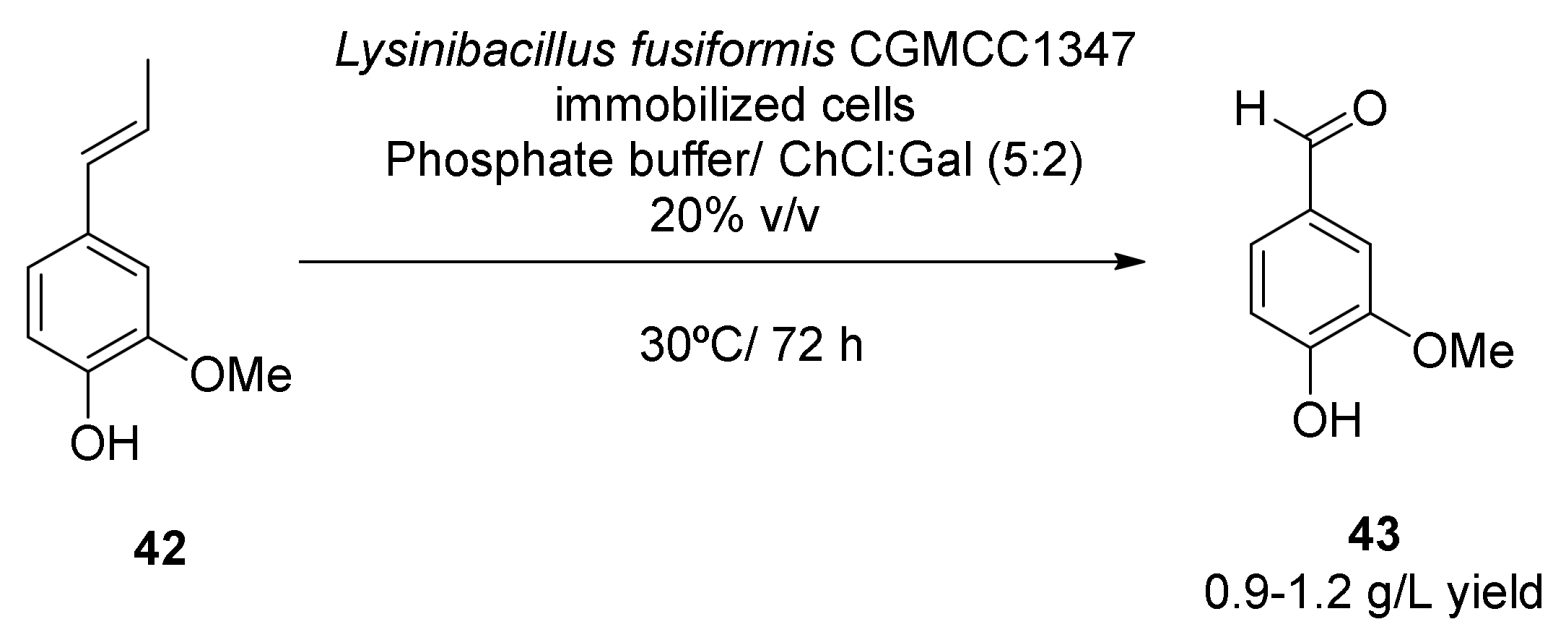
Oxidation of isoeugenol to vanillin catalyzed by *Lysinibacillys fusiformis* CGMCC1347 cells in buffer containing DES.

In 2017, a set of 45 eutectic solvents (24 DESs and 21 NADESs) were tested as cosolvents on the biotransformation of isoeugenol (**42**) into vanillin (**43**) using *Lysinibacillus fusiformis* CGMCC1347 cells (Scheme 18) [90]. Almost all the (NA)DES at 1% *v*/*v* in water enhanced the bioconversion because they increased the bacterial cell membrane permeability for the lipophilic substrate. Those DES containing choline acetate (ChAc) usually led to better yields than those composed of ChCl, being possible to obtain increases of 142% regarding the reaction in only water. When the oxidations were performed with 20% *v*/*v* of (NA)DES, half of these solvents improved the product yield, being observed a relationship between the hydrogen bond donor of the DES and the yield in the order: organic acids < alcohols < sugars. Whole-cells were immobilized on poly(vinyl alcohol)-alginate (PVA) beads. The biotransformation was conducted for 13 times during 72 h at 30 °C in phosphate buffer pH 7.0 containing 20% *v*/*v* choline chloride:galactose [ChCl:Gal (5:2)] without loss of the system activity, which represents a promising result for further development.

**Scheme 19:**
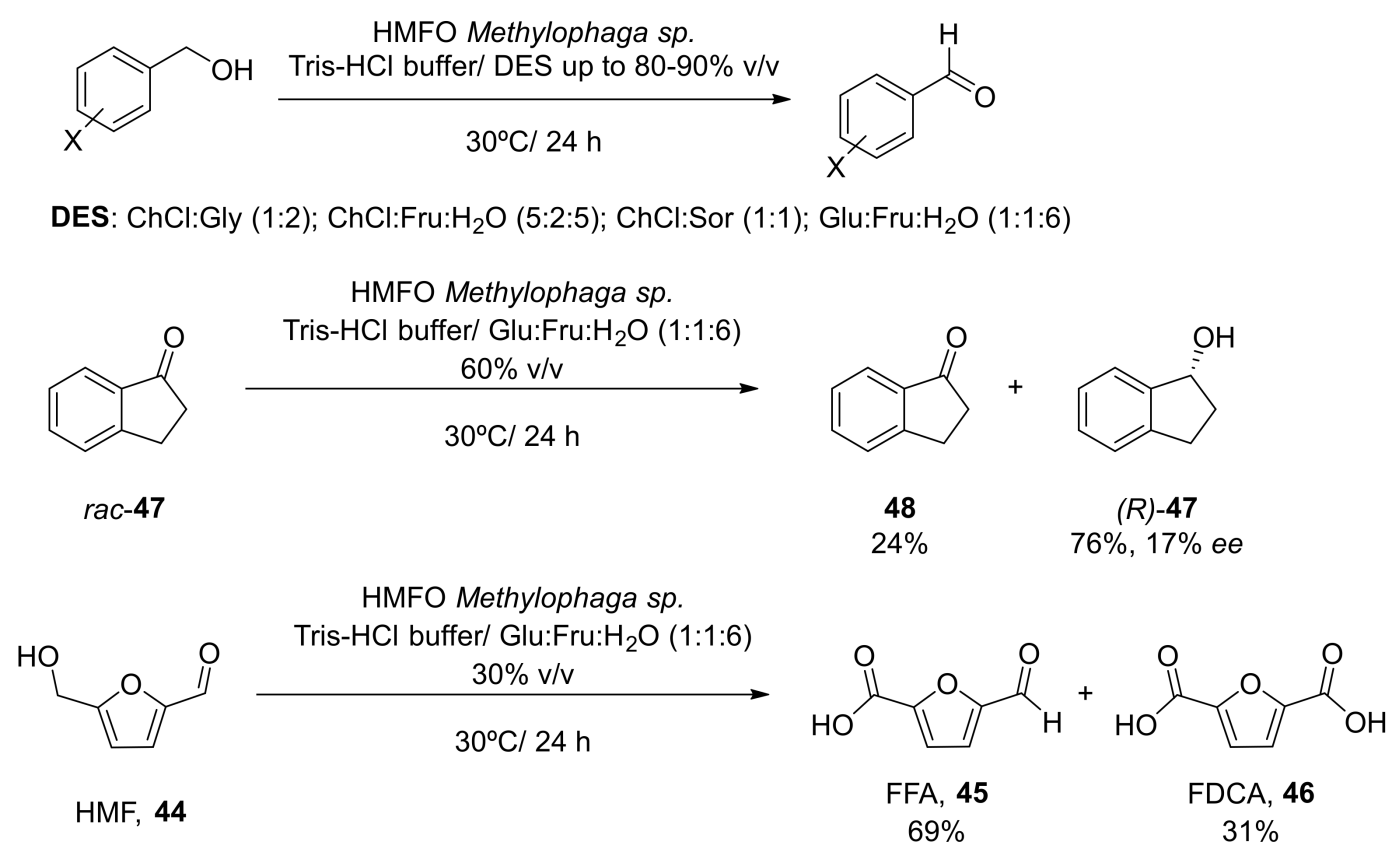
Application of hydromethylfurfural oxidase from *Methylophaga* sp. in oxidative processes using a glucose-fructose-based NADES.

Deep eutectic solvents have also been employed as cosolvents in biotransformations catalyzed by flavin-dependent oxidases. These enzymes are able to perform the oxidation of different substrates using molecular oxygen as mild oxidant [91]. Recently, several NADESs have been tested with hydroxymethylfurfural oxidase (HMFO) from *Methylophaga* sp., which catalyzes the valuable oxidation from hydroxymethylfurfural (HMF, **44**) to 5-formylfuran-2-carboxylic acid (FFA, **45**) and to the valuable furan-2,5-dicarboxylic acid (FDCA, **46**), a chemical platform for the production of several biobased compounds, through a sequential oxidation process [92]. Initial experiments were performed using the purified enzyme in the biocatalyzed oxidation of different aromatic alcohols to the corresponding aldehydes, as shown in Scheme 19 [93], being observed than in 60% *v*/*v* of the choline choline-sugar DESs as ChCl:Sor (1:1), ChCl:Glu:H_2_O (5:2:5) or ChCl:Fru:H_2_O (5:2:5), or even the NADES formed by the sugars Glu:Fru:H_2_O (1:1:5) at 60 or even 80% *v*/*v*, much higher conversions were obtained respecting the biooxidations in neat buffer Tris/HCl pH 8.0. In addition, much higher substrate concentrations can be used in the bioxidations in presence of DESs, being possible to work at 100 or even 200 mM. HMFO was also found to oxidize 1-indanol (**47**) to 1-indanone (**48**) in a moderately enantioselective manner when using the NADES Glu:Fru:H_2_O (1:1:6), recovering the remaining (*R*)-enantiomer of **46** with 17% *ee* at a 24% conversion. This cosolvent was studied in the oxidation of HMF to FDCA, being observed at 30% *v*/*v* DES a complete conversion into a mixture of FFA (69%) and FDCA (31%), a much better result than the obtained in the biocatalyzed oxidation in buffer alone. Higher contents of the NADES in the reaction medium led to lower conversions for this biotransformation, but it is possible to completely oxidize HMF at 60% *v*/*v* of ChCl:Glu:H_2_O (5:2:5).

The effect of several ChCl and ethylammonium chloride (EAC) based DESs on the heme-dependent enzymes cytochrome c from equine heart (cyt c) and horseradish peroxidase (HRP) has been analyzed [94]. After observing the beneficial effect on both enzymes stability of mixtures DES-aqueous buffer for both enzymes, the decolorization activity of cyt c was carried out using pinacyanol chloride, a valuable industrial dye, as substrate. When the reactions were performed on EAC:EG (1:1.5) or EAC:U (1:1.5) in 50% *v*/*v* increased this decoloration activity by 3.3 times respecting buffer alone. The reusability of the both DESs was studied for this reaction when cyt c was immobilized onto Celite, being observed that the eutectic solvents can be reused three times without significant loss in enzymatic activity.

**Scheme 20:**
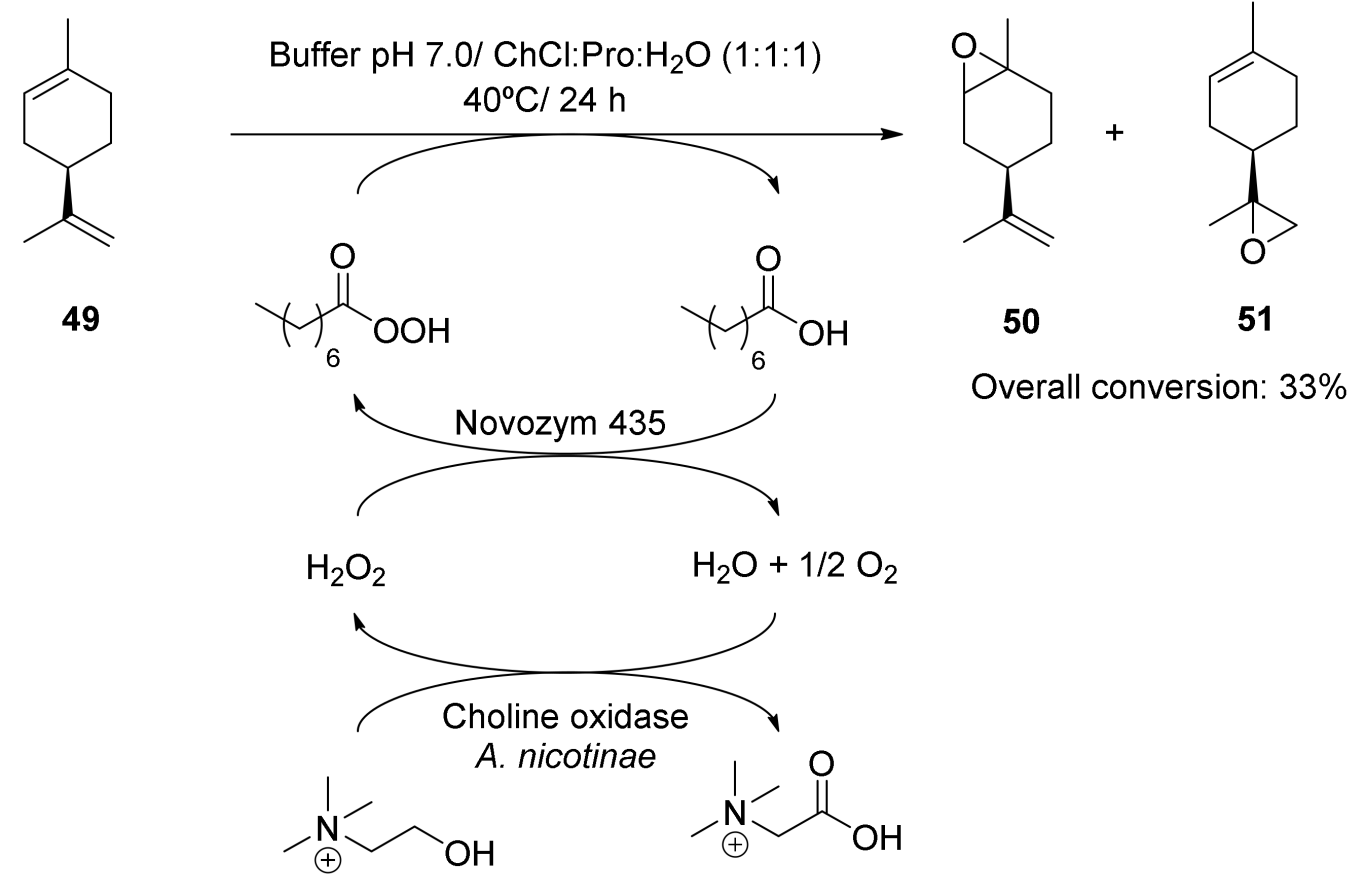
Chemoenzymatic cascade employing ChCl:PD:H_2_O (1:1:1) as cosolvent and ChOx/CalB for the epoxidation of limonene obtained from orange peel wastes.

In 2019, a set of NADEs has been employed with a triple purpose in the valorization of orange peel wastes (Scheme 20) [95]. These compounds have been employed as solvents for the extraction of limonene (**49**) from these wastes, as cosolvents in the biocatalyzed epoxidation of limonene to compounds **50** and **51**, and finally as sacrificial electron donor for the in situ formation of the oxidant hydrogen peroxide. Initially, different NADESs were tested in the extraction of limonene from orange peel, achieving efficient extractions in presence of different ChCl based DES, including ChCl:1,2-propanediol(PD):H_2_O (1:1:1). The hydrogen peroxide formation was catalyzed by the choline oxidase (ChOx) from *Arthrobacter nicotinae* overexpressed in *E. coli*. This enzyme was able to oxidize choline chloride into betaine with the concomitant formation of H_2_O_2_ as byproduct, so the DES can be employed as source of one of the co-substrates of the system. Hydrogen peroxide formation was optimized in mixtures ChCl:PD:H_2_O (1:1:1) in 25–75% *v*/*v* ratio with aqueous buffer, at temperatures between 30–40 °C and was dependent on the ChOx concentration. Finally, H_2_O_2_ was able to react with different organic acids to generate the corresponding peracids in a process catalyzed by a hydrolase. The peracid formed was able to oxidize limonene (**49**) to limonene epoxides (**50** and **51**) in a catalytic cascade. The best biocatalyst for the peracid formation was the lipase B from *Candida antarctica* (CalB), a widely employed hydrolase immobilized as the Novozym 435 preparation. When the catalytic cascade was performed at the optimized conditions, employing a 1:1 mixture of the DES and pH 7.0 buffer at 40 °C, using octanoic acid as the source of peracid and the enzymatic system ChOx/CalB, it was possible to obtain a mixture of limonene monoepoxide and limonene diepoxide in 33% conversion after 24 h when starting from different orange peel wastes.

**Scheme 21:**
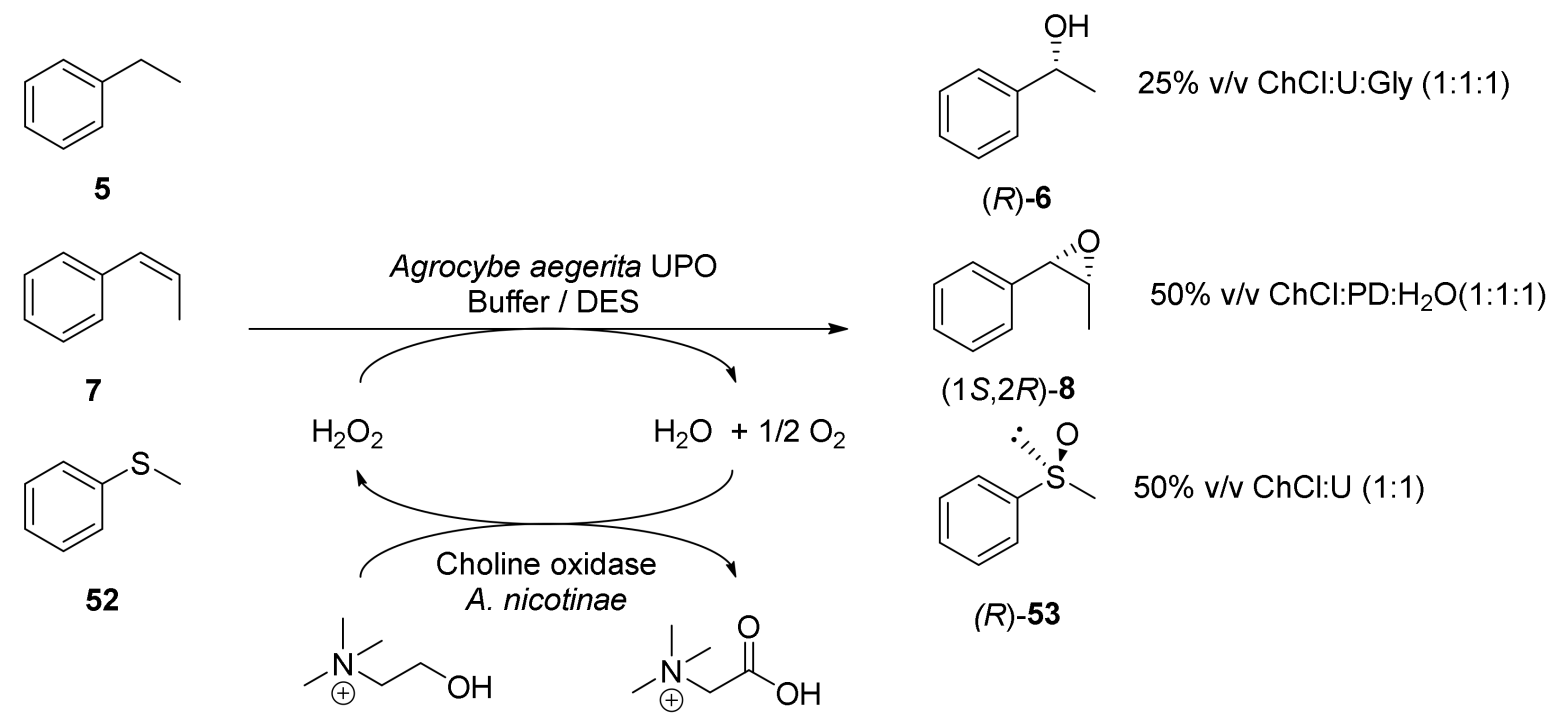
General process for the bienzymatic reaction of ChOx from *A. nicotinae* as H_2_O_2_ generator with r*Aae*UPO to perform oxidative processes in aqueous buffer mixtures containing different DESs.

Peroxygenases have also been employed using DESs as cosolvents. Among the different examples of hydrogen peroxide in situ generation described, choline oxidase ChOx has been also employed to this purpose. Thus, the choline chloride-based DESs can be employed as solvents and as electron donors. This oxidase has been combined with the peroxygenase from *Agrocybe aegerita* in order to perform effective biotransformations, including the hydroxylation of ethyl benzene (**5**) and the epoxidation of *cis*-β-methylstyrene (**7**) (Scheme 21) [96]. Reactions cannot be developed in neat NADES, as no product formation was observed, being required some amount of buffer to ensure a proper enzymatic activity. After optimization of different reaction parameters, it was observed that both oxidations present their highest performance at different conditions. For **5** hydroxylation, the best result was achieved with 25% *v*/*v* of ChCl:U:Gly (1:1:1), while for the epoxidation the best conditions were found with 50% *v*/*v* of ChCl:PD:H_2_O (1:1:1). Products were obtained in all the cases with optical purities higher than 95%, and despite the fact that there is still necessary a further optimization of this biocatalytic procedure, it is possible to achieve high catalytic turnovers for r*Aae*UPO in this reaction medium.

In a further development of the application of peroxygenases in DES media, r*Aae*UPO has been employed in the enantioselective sulfoxidation of thioanisole (**52**) in combination with ChOx, using choline chloride-based DES as solvents and as electron donors (Scheme 21) [97]. In all the reactions tested, (*R*)-methyl phenyl sulfoxide (**53**) was recovered with complete enantioselectivity, with conversions around 60%. In order to achieve some sulfoxidation activity, at least a 25% *v*/*v* of aqueous solvent was required in the reaction media. Best results were observed in urea-based DES, being possible to obtain the highest conversions when using ChOx concentrations of 3 μM and the DESs ChCl:U (1:1) or ChCl:U:Gly (1:1:1) in concentrations of 50% *v*/*v*.

Table 4 summarizes the application of NADES in biooxidation processes in presence of different types of biocatalysts.

## 5. Conclusions and Future Perspectives

Biocatalysis has evolved in the last years as a valuable and green technology for the preparation of high added value chemicals. Among all the biocatalysts employed in organic synthesis, redox enzymes present a great interest for synthetic chemists. They catalyze all types of reductive and oxidative processes under mild reaction conditions using of mild oxidants or reductants, achieving in general excellent selectivities, while reducing the environmental risks derived from the classical chemical oxidants/reductants. The use of aqueous buffers as reaction medium for this type of processes presents some advantages, but unfortunately, low substrates concentrations are attainable and low productivity are generally achieved, thus preventing for the application of these type of processes at industrial scale. For this reason, a set of alternative green reaction media have been applied in the biocatalyzed redox reactions, with the aim of maintaining the benefits inherent to biocatalytic reactions in terms of selectivity and environmental issues, while increasing the substrate loadings and process productivities.

Due to the characteristics of redox enzymes, which require in most cases external cofactors to develop their activity, or the use of whole-cells systems (with the limitations that they present), the application of green reaction media is not as widespread as for hydrolases, which are really robust biocatalysts able to work in a wide set of non-conventional reaction media. Thus, until nowadays, three approaches using green reaction media have been performed.

The use of neat conditions, in which water in almost completely suppressed from the reaction, and the development of reactions in mixtures aqueous buffers with biobased solvents such as 2-MeTHF and CPME, has been explored with some examples, especially for reductive enzymes, but these two approaches still required for much further investigation in order to develop better redox sustainable processes.

In contrast, the application of NADESs as organic cosolvents in reductive and oxidative processes is a field that has been widely studied since the seminal works six years ago. In general, bioreductions catalyzed by ADHs, in most cases employing whole-cells biocatalysts, in different amounts (NA)DESs, have experienced a great development in the last few years. Some examples have been shown in which the modification of the reaction medium allowed to reverse the enzymatic enantiopreference, whereas in other cases, the presence of these green solvents afforded higher activities and/or selectivities, due to the effect of the neoteric solvent in the cell membrane permeability. In the recent times, (NA)DESs are starting to be employed not only as the reaction cosolvent, but also as a reagent that can be consumed in the reaction for the cofactor regeneration or even for the generation of one reactive of the biocatalyzed reaction. This is an interesting field that has to be still developed, by increasing the scope of NADESs in this type of reactions. But in general, we have to consider that still further research is required in order to rationalize the effect of these neoteric solvents in the enzymatic conformation with the aim of obtaining a better understanding for these reactions. Two final aspects need to be consider when employing NADES as cosolvents in redox biocatalyzed processes. First, NADES are good solvents with several applications in extraction processes [98,99,100], so sometimes the recovery of the final product from the reaction medium can present some issues, a question to be taken into account for process designing. Finally, NADES can be recycled from the reaction media and used for several times, another aspect to consider. Until nowadays, not many examples of DES recycling in catalytic processes have been performed [101,102], being an area of great interest for organic chemists that has to be developed.

## Figures and Tables

**Table 1:** Overview of biotransformations catalyzed by oxidorreductases in the presence of different proportions of CPME and 2-MeTHF.

Biocatalyst	Solvent (% *v*/*v*)	Yield (%)	*ee* (%)	Reference
Enoate reductases	CPME 20% ***v*/*v***	<10	0–99	[53]
Imine reductases	CPME 90% ***v*/*v***	36–96	46–99	[58]
ADH	CPME 10% ***v*/*v***	>90	>90	[59]
ADH	CPME 5% ***v*/*v***	99	99	[60]
Monooxygenase + ADH	CPME 5% ***v*/*v***	27	-	[61]
Alcohol and glycerol dehydrogenases	2-MeTHF 5% ***v*/*v***	75–99	>99	[64]
*Saccharomyces cerevisiae* YOL151W Reductase	2-MeTHF 3% ***v*/*v***	99	>99	[65]

**Table 2:**
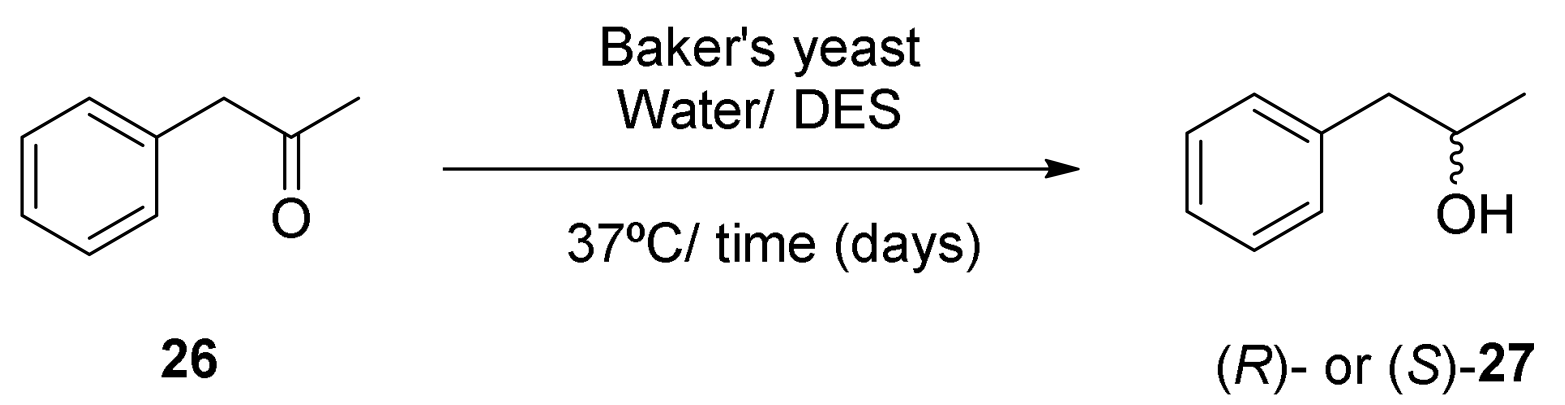
Baker’s yeast catalyzed bioreduction of phenylacetone (**26**) in presence of different choline chloride-based DESs containing D-fructose or glycerol.

Entry	Solvent	Time (days)	Yield (%)	*ee* (%)	Config.
1	Water	1	88	96	*S*
2	Water + ChCl:Fru (3:2) 60% *v*/*v*	5	31	78	*S*
3	Water + ChCl:Fru (3:2) 90% *v*/*v*	5	14	60	*R*
4	Water + ChCl:Gly (1:2) 50% *v*/*v*	6	88	88	*S*
5	Water + ChCl:Gly (1:2) 80% *v*/*v*	6	44	60	*R*
6	Water + ChCl:Gly (1:2) 90% *v*/*v*	6	36	96	*R*

**Table 3:** Examples of optically active aliphatic and aromatic alcohols that can be obtained employing ADHs in presence of NADES as cosolvents.

Product	ADH	NADES (% *v*/*v*)	Yield (%)	*ee* (%)	Ref.
(*S*)-1-phenylethanol	*Ralstonia* sp. *E. coli* cells	ChCl:Gly (1:2) 80% *v*/*v*	90 ^(a)^	98	[79]
(*S*)-1-phenyl-1-propanol	*Ralstonia* sp. *E. coli* cells	ChCl:Gly (1:2) 80% *v*/*v*	90 ^(a)^	82	[79]
(*R*)-2-octanol	*Acetobacter pasteurianus* GIM1.158 cells	ChCl:EG (1:1) 10% *v*/*v*	96	99	[80]
(*S*)-4-chloro-3-hydroxybutanoate	*E. coli* CCZU-T15 cells	ChCl:Gly (1:2) 12.5% *v*/*v*	93	>99	[81]
(1*S*,3*S*)-α’-1-hydroxyethyl-γ-butyrolactone	*Candida viswanathi* AM120 cells	ChCl:Gly (1:2) 10% *v*/*v*	81	76	[82]
(*R*)-1-phenyl-1,2-ethanodiol	*Kurthia gibsonii* SC0312 cells	ChCl:BD (1:4) 2% *v*/*v*	80	>99	[83]
(*S*)-3,5-bistrifluoromethyl- phenyl ethanol	*Rhodococcus erythropolis* XS1012 cells	ChCl:U (1:1) 1% *w*/*v*	81	>99	[84]
(*R*)-3,5-bistrifluoromethyl- phenyl ethanol	*Trichoderma asperellum* ZJPH0810 cells	ChCl/GSH (1:1) 1% *v*/*v*	91	>99	[85]
(*S*)- (3,4-dimethylphenyl)ethanol	Carrot roots cells	ChCl:Gly (1:2) 20% *v*/*v*	50	83	[86]
(*S*)-2-octanol	Lyophilized ADH-T	ChCl:Glu (1.5:1) 10% *v*/*v*	85	>99	[87]
(*R*)-1-phenyl-1-propanol	KRED P1-A04	ChCl:Gly (1:2) 50% *v*/*v*	95	>99	[88]
(*S*)-1-(4-bromophenyl)-1-propanol	KRED P2-H07	ChCl:Gly (1:2) 50% *v*/*v*	90	>99	[88]

^(a)^ These values refer to conversion, not to isolated yield.

**Table 4:** Oxidations catalysed by biocatalysts in different NADES.

Biocatalyst	NADES (% *v*/*v*)	Reference
*Acetobacter* sp. CCTCC M209061 immobilized cells	ChCl:Gly (1:2) 10% *v*/*v*	[89]
*Lysinibacillus fusiformis* CGMCC1347 cells	ChCl:Gal (5:2) 20% *v*/*v*	[90]
HMFO from *Methylophaga* sp	Glu:Fru:H_2_O (1:1:5) 60–80% *v*/*v*	[93]
Novozym 435 + ChOx from *Arthrobacter nicotinae*	ChCl:Pro:H_2_O (1:1:1) 25–75% *v*/*v*	[95]
*rAae*UPO + ChOx from *Arthrobacter nicotinae*	ChCl:U:Gly (1:1:1) 25% *v*/*v*	[96]
*rAae*UPO + ChOx from *Arthrobacter nicotinae*	ChCl:U (1:1) 50% *v*/*v*	[97]
